# Genome-wide identification and comprehensive analysis of WRKY transcription factor family in safflower during drought stress

**DOI:** 10.1038/s41598-023-44340-y

**Published:** 2023-10-07

**Authors:** Xianming Song, Xianfei Hou, Youling Zeng, Donghai Jia, Qiang Li, Yuanguo Gu, Haocui Miao

**Affiliations:** 1 Present Address: Economic Crop Research Institute, Xinjiang Academy of Agricultural Sciences, Urumqi, 830091 China; 2 Present Address: Xinjiang Key Laboratory of Biological Resources and Genetic Engineering, College of Life Science & Technology, Xinjiang University, Urumqi, 830046 China

**Keywords:** Plant sciences, Plant molecular biology

## Abstract

The WRKY family is an important family of transcription factors in plant development and stress response. Currently, there are few reports on the WRKY gene family in safflower (*Carthamus tinctorius* L.). In this study, a total of 82 *CtWRKY* genes were identified from the safflower genome and could be classified into 3 major groups and 5 subgroups based on their structural and phylogenetic characteristics. The results of gene structure, conserved domain and motif analyses indicated that *CtWRKYs* within the same subfamily maintained a consistent exon/intron organization and composition. Chromosomal localization and gene duplication analysis results showed that *CtWRKYs* were randomly localized on 12 chromosomes and that fragment duplication and purification selection may have played an important role in the evolution of the WRKY gene family in safflower. Promoter cis-acting element analysis revealed that the *CtWRKYs* contain many abiotic stress response elements and hormone response elements. Transcriptome data and qRT-PCR analyses revealed that the expression of *CtWRKYs* showed tissue specificity and a strong response to drought stress. Notably, the expression level of the *CtWRKY55* gene rapidly increased more than eightfold under drought treatment and rehydration, indicating that it may be a key gene in response to drought stress. These results provide useful insights for investigating the regulatory function of the *CtWRKY* gene in safflower growth and development, as well as identifying key genes for future molecular breeding programmes.

## Introduction

Transcription factors (TFs) play an important role in plant growth and development and biotic and abiotic stress responses by regulating the transcription of downstream target genes through interaction with cis-acting elements. Since the first extraction of the WRKR gene *SPF1* from *Pomoea batatas* in 1994, researchers have identified the corresponding *WRKY* genes from *Acer truncatum*, *Taraxacum kok-saghyz* Rodin, *Chrysanthemum lavandulifolium*, *Linum usitatissimum* L. and *Zea mays*^1–5,8^. Hu Wenjing et al. found that the conserved domain of WRKY family members consists of 60–70 amino acids, including a highly conserved seven-phthalide sequence (WRKYGQK) and a zinc finger structure (C_2_H_2_ or C_2_HC)^5^. Researchers discovered that members of the WRKY gene family could be divided into Groups I, II and III based on the number of conserved structural domains and the type of zinc finger structures, and group II could be further divided into five subgroups, IIa, IIb, IIc, IId and IIe, based on differences in amino acid sequences^6,8^. Among them, members of Group I have two conserved WRKY structural domains, and members of groups II and III have one conserved WRKY structural domain; members of Groups I and II have C–X_4_-_5_C–X_23-24_–H–X_1_–H (C_2_H_2_) zinc finger structures, and members of group III have C–X_5_–_7_–C–X_23_–_38_–H–X_1_–C (C_2_HC) zinc finger structures^6^. WRKY transcription factors can specifically bind to cis-acting elements in the promoter regions of target genes to regulate the expression of downstream genes and enhance plant resistance to abiotic stresses such as low temperature, drought and salt, as well as to pathogenic bacteria^4,6,7,7,8^. For example, suppression of *GhWRKY21* has been shown to improve drought tolerance in cotton, although *GhWRKY21* plays a negative role in the drought response in cotton^9^. *MaWRKY80* overexpression results in improved phenotypic morphology, enhanced survival, reduced water loss rate, and lower malondialdehyde (MDA) levels compared to the wild-type (WT) under drought stress. Notably, the leaves of transgenic *MaWRKY80 Arabidopsis* exhibited lower reactive oxygen species (ROS) levels than WT leaves under drought stress^10^. *AtWRKY11* and *AtWRKY70* can coordinate their resistance to Bacillus through jasmonic acid (JA) and salicylic acid (SA) signalling pathways, and their overexpression can enhance drought tolerance and promote seed germination and root growth under drought stress in *Arabidopsis*^11^. The sorghum WRKY transcription factor *SbWRKY30* is mainly expressed in leaves and roots and is induced by drought stress. Heterologous expression of *SbWRKY30* in *Arabidopsis* and rice confers drought tolerance by altering root architecture^12^. Through the ABA signalling pathway, *ZmWRKY79* can improve drought tolerance in maize^13^. The *ClWRKY20* expression level rose in response to treatment with salinity, drought, and phytohormones (ABA, ET, and SA). Transgenic *Arabidopsis* exhibited heightened sensitivity to ABA during seed germination and under low temperatures and salinity upon overexpression of *ClWRKY20*^14^. Additionally, *WRKY* genes, which respond to drought stress, have also been identified in other crops, providing evidence that WRKY transcription factors are crucial in responding and adapting to drought stress^15–18^.

Safflower (*Carthamus tinctorius* L.) is a special economic crop utilized in medicinal materials, oil, dyes and feed. It has the characteristics of strong environmental adaptability, resistance to high temperature and drought, and a short growth cycle^19,20^. The Xinjiang Uygur Autonomous Region is the main safflower-producing area in China. This perennially utilized area accounts for more than 80% of the area and yield of safflower in China^21^. Xinjiang belongs to the “temperate continental climate” zone, with a large temperature range, sufficient sunshine (the annual sunshine time reaches 2 500 to 3 500 h), low precipitation and a dry climate. Safflower has strong stress resistance, but its quality and yield are still affected by drought, high temperature and scarce rainfall^22–24^. In 2021, Wu Zhihua et al. sequenced the safflower genome, which provided us with a helpful foundation to further analyse the gene function of safflower^23^.

Safflower represents an important economic crop. Studies have shown that multiple transcription factor families have been found in safflower, such as MYB, bHLH and bZIP transcription factor families^25–27^. Nevertheless, the molecular mechanism governing drought tolerance regulation in safflower remains unknown. Furthermore, there has been no account of the genome-wide identification and functional analysis of the WRKY gene family in safflower. In this study 82 *CtWRKY* genes were identified from the safflower genome and their physicochemical properties, phylogeny, chromosomal location, gene structure, conserved motifs, cis-acting elements, collinearity and protein interaction networks were comprehensively analysed by bioinformatics. Transcriptome data and qRT-PCR were used to analyse *CtWRKY* gene expression patterns in different tissues and under drought stress and rewatering treatments. This study provides valuable insights for investigating the regulatory function of the *CtWRKY* gene in safflower growth and development, as well as identifying key genes for future molecular breeding programmes.

## Results

### Identification and physicochemical property analysis of WRKY gene family members in safflower

Using the protein sequence of the *Arabidopsis* WRKY gene family and the WRKY domain hidden Markov model (HMM) file (PF03106), 83 *CtWRKY* gene family members were preliminarily identified from the safflower genome by BLAST alignment, and then incomplete sequences and candidate sequences without corresponding domains were eliminated. A total of 82 *CtWRKY* gene family members were identified from the safflower genome, which were named according to their positions on the 12 safflower chromosomes, namely, *CtWRKY1*–*CtWRKY82* (Table S1).

As shown in Table S1, the coding sequences (CDS) length of *CtWRKY* genes ranged from 504 (*CtWRKY78*) to 4 011 bp (*CtWRKY30*). The length of CtWRKY proteins varied from 167 (*CtWRKY78*) to 1 366 bp (*CtWRKY30*) and the average protein sequence length was 379 residues. The molecular weight of the proteins was 19 130.99 Da (C*tWRKY78*)–150 954.83 Da (*CtWRKY30*), with an average of 42 203.72 Da. The isoelectric point was 4.92 (*CtWRKY38*)–10.15 (*CtWRKY24*). The total average hydrophilicity index revealed that CtWRKY proteins were hydrophilic proteins. Subcellular localization prediction showed that most *CtWRKY* genes were in the nucleus, and a few *CtWRKY* genes were in the peroxisomes (*CtWRKY6*, − *8* and − *48*), chloroplasts (*CtWRKY33*), cytoplasm (*CtWRKY52*) and mitochondria (*CtWRKY75*).

### Phylogenetic analysis of the WRKY gene in safflower

**Figure 1:**
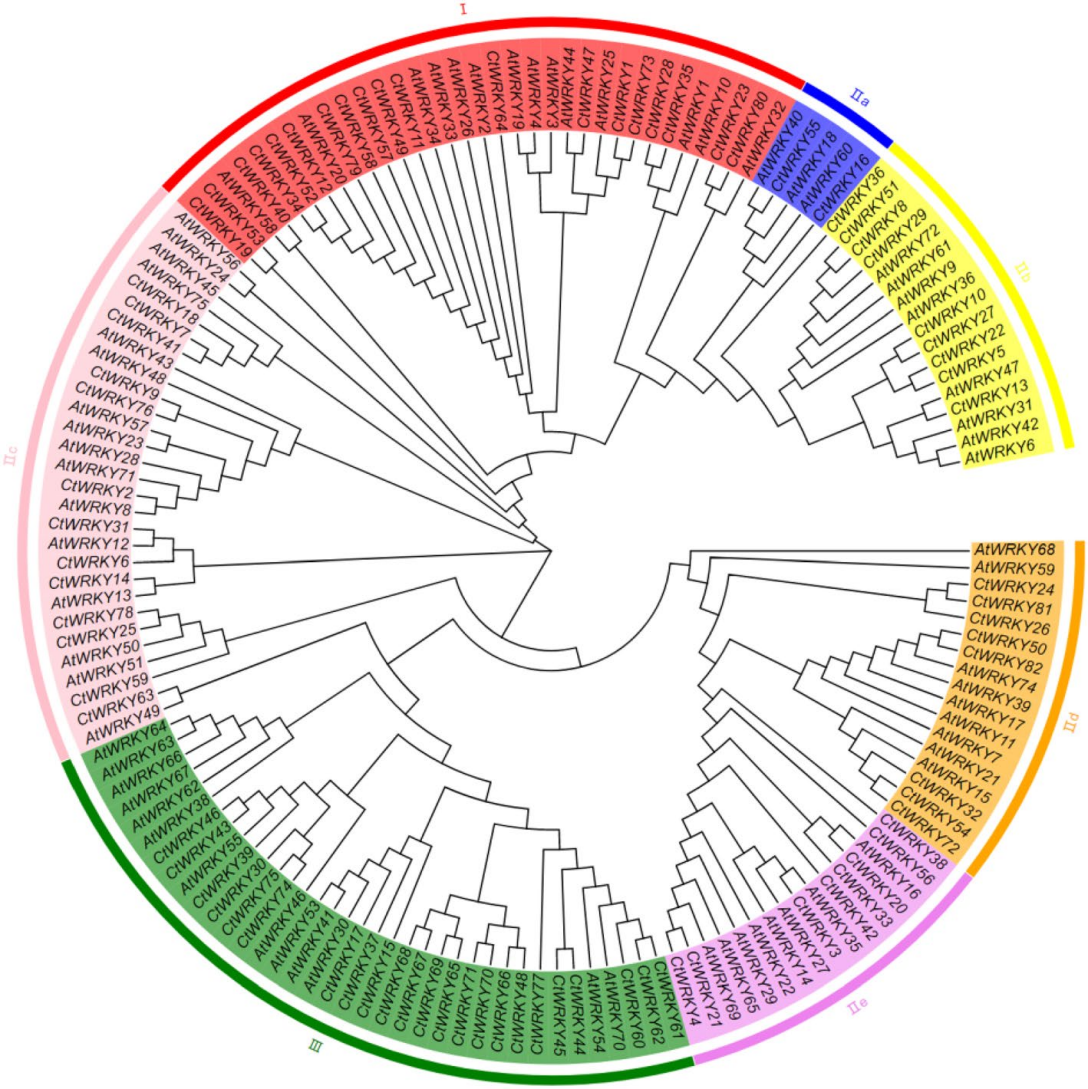
Phylogenetic analysis of safflower and *Arabidopsis* WRKY proteins. An unrooted neighbour-joining (NJ) phylogenetic tree was constructed with WRKY domains of WRKY proteins from safflower and *Arabidopsis* using MEGA7.0 with a bootstrap of 1000. Seven major clades are indicated: I, IIa, IIb, IIc, IId, IIe, and III with different coloured areas.

Using the *Arabidopsis* WRKY gene family as a reference, the phylogenetic tree of safflower and *Arabidopsis* WRKY protein sequences was constructed by the neighbour-joining method (Fig. 1). The results revealed that CtWRKY proteins were divided into three groups (I–III) according to the grouping of AtWRKY gene family members, and group II was divided into five subgroups: IIa, IIb, IIc, IId, and IIe. Multiple sequence alignment revealed that the heptapeptide sequence (WRKYGQK) and zinc finger domains (C_2_H_2_ and C_2_HC) were conserved in each group. In addition, three heptapeptide variants were found: WRKYGKK (*CtWRKY25*, − *59* and − *78*) in group IIc, WKKYGEK (*CtWRKY24*, − *26* and − *81*) in group IId and WRKYGHK (*CtWRKY60*) in group III (Fig. S1). Group I included 17 *CtWRKY* members, all of which had 2 WRKY conserved domains and a C_2_H_2_ zinc finger structure. Group II included 42 *CtWRKY* members, all of which had a WRKY conserved domain and a C_2_H_2_-type zinc finger structure. They were further divided into 5 subgroups according to their genetic relationship and structure: IIa (2 proteins), IIb (9 proteins), IIc (15 proteins), IId (8 proteins) and IIe (8 proteins) subgroups. Group III included 23 *CtWRKY* members, all of which had a WRKY conserved domain and a C_2_HC zinc finger structure.

### Analysis of the chromosomal localization, gene structure and conserved motif of the WRKY gene in safflower

**Figure 2:**
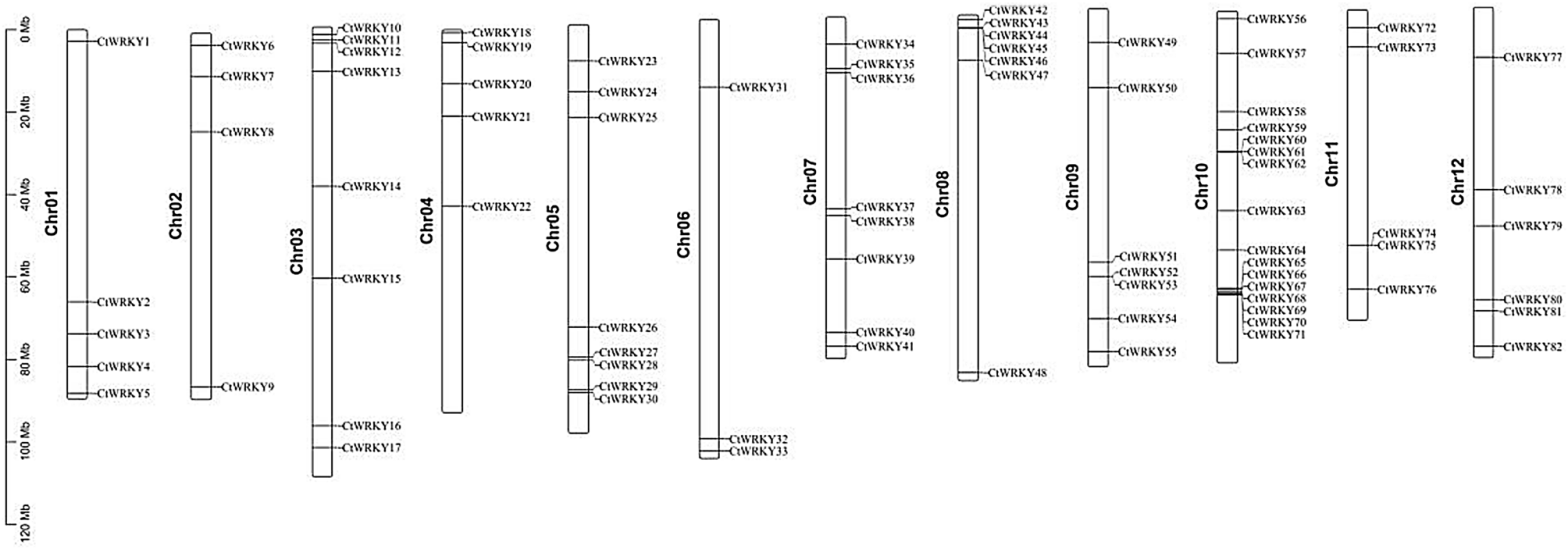
Chromosomal localization of the WRKY gene in safflower. A total of 82 *CtWRKY* genes were mapped to the ten safflower chromosomes with an uneven distribution. The gene name on the right side of each chromosome corresponds to the approximate location of each *CtWRKY* gene. The scale on the left is in megabases.

The distribution of the *CtWRKY* gene on safflower chromosomes was analysed with TBtools software (Fig. 2). The results showed that 82 *CtWRKY* genes were unevenly distributed on the 12 safflower chromosomes. The highest number of *CtWRKY* genes was found on chromosome 10 (16 genes), and the lowest number of *CtWRKY* genes was found on chromosome 6 (3 genes).

**Figure 3:**
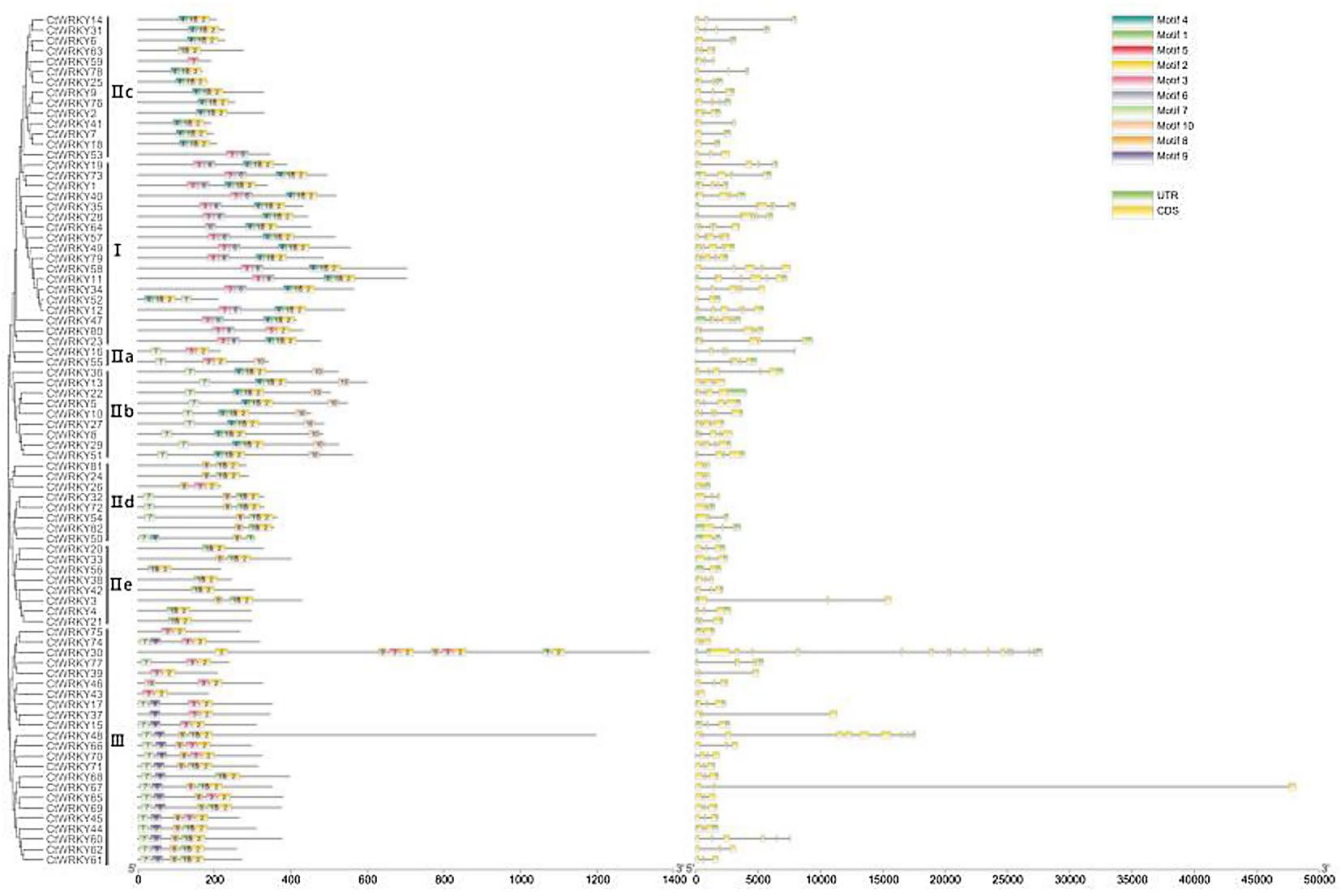
Phylogenetic tree, gene structure and conserved motif of the WRKY gene in safflower. The evolutionary tree was constructed from the CtWRKY protein sequences using MEGA 7.0 software with the neighbour-joining method. The structure of all 82 *CtWRKY* genes was obtained by TBtools software. The conserved motifs of CtWRKY proteins were analysed using the MEME tool.

The diversity of gene structure promotes the evolution of the gene family. To further understand the evolutionary process of the CtWRKY gene family, the structure and conserved motifs of the *CtWRKY* gene were analysed in combination with the CtWRKY protein phylogenetic tree (Fig. 3). Ten conserved motifs were identified in CtWRKY proteins using the MEME tool, and 82 CtWRKY proteins were found to have specific conserved domains (Fig. S2 and Table S2). As shown in Fig. 3, most CtWRKY family members in the same group or subgroup shared similar conserved motifs, but there were some differences between groups. Furthermore, members of the same group or subgroup had specific conserved motifs. For example, motif 6 was present only in Group I members, motif 10 was present only in group IIa and IIb members, motif 9 was present only in group III members, and the group III member CtWRKY30 protein contained two motif 3 s, which are rare in other species. These results suggest that these conserved motifs may play different roles in gene evolution and function. Additionally, almost all members of the CtWRKY family contain a WRKYGQK heptapeptide sequence (motif 1 or motif 3), indicating that WRKYGQK is the core conserved motif of the CtWRKY gene family.

According to the results of gene annotation, the exon‒intron coding sequence structure of the *CtWRKY* gene was analysed (Fig. 3). The results revealed that CtWRKY family members had 2–17 exons, and a few members had no UTR. Among them, most *CtWRKY* genes contained 4 exons (2 introns), accounting for 48.78% (40/82) of all *CtWRKY* genes. The *CtWRKY48* gene contained 10 exons, and the *CtWRKY30* gene contained 17 exons. Consistent with conserved motif analysis, CtWRKY members in the same group or subgroup had similar gene structures and a high degree of conservation, indicating functional similarity between different members.

### Analysis of cis-acting elements and collinearity of the WRKY gene in safflower

**Figure 4:**
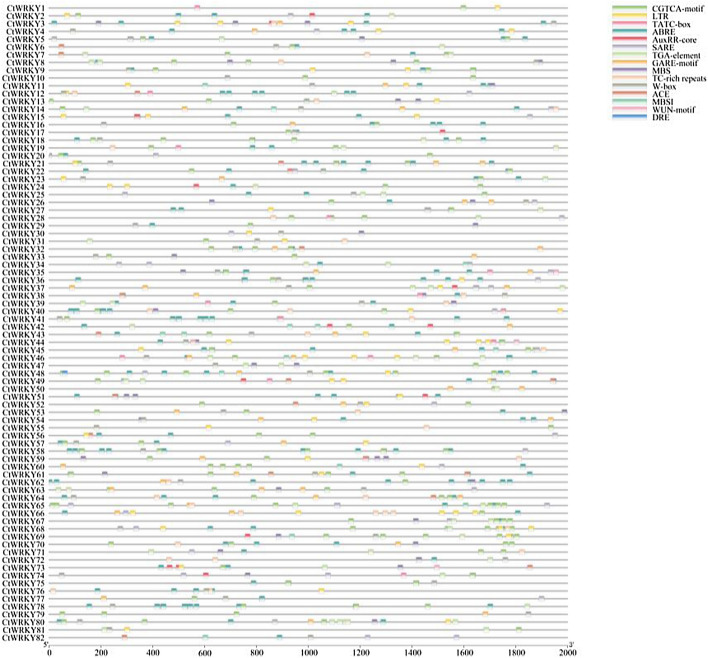
Distribution of cis-acting elements in the promoter region of the WRKY gene in safflower. The putative DRE, LTR, MBS, WUN motif, W-box, TATC box, GARE motif, AuxRR core, TGA element, CGTCA motif, TGACG motif, ABRE, SARE and MBSI core sequences are represented in different colours. DRE: cis-acting elements involved in dehydration, low temperature and salt stress responses; ABRE: cis-acting element involved in abscisic acid responsiveness; LTR: cis-acting element involved in low-temperature responsiveness; MBS: MYB binding site involved in drought inducibility; TGACG-motif and CGTCA-motif: cis-acting regulatory element involved in MeJA responsiveness; TC-rich repeats: cis-acting element involved in defence and stress responsiveness; TATC-box and GARE-motif: gibberellin-responsive element; W box: elicitation; wounding and pathogen responsiveness; AuxRR-core and TGA-element: auxin-responsive element; SARE: cis-acting element involved in salicylic acid responsiveness; MBSI: MYB binding sites involved in the regulation of flavonoid biosynthesis genes.

The 2 000 bp promoter sequence of the *CtWRKY* gene was extracted, and its cis-elements were analysed using the PlantCARE database to explore the potential function of the *CtWRKY* gene in abiotic stress response (Fig. 4). The results revealed that a total of 14 cis-acting elements associated with plant hormones and stress responses were found in the promoter region of the *CtWRKY* gene, including 1 light response element (ACE); 6 abiotic stress response elements (DRE, LTR, MBS, W-box, WUN motif and TC-rich repeats); and 7 hormone response elements (SARE, ABRE, TATC box, TGA element, AuxRR core, CGTCA motif and GARE motif). As shown in Figure S3, the promoter sequence of the *CtWRKY* gene contains the most cis-acting elements: the CGTCA motif (methyl jasmonate response element) and ABRE (abscisic acid response element). Approximately 68 genes contained these two elements. The promoter regions of 60 *CtWRKY* genes contained W-box elements, indicating that these genes may be regulated by other WRKY transcription factors or by themselves. Furthermore, 39 *CtWRKY* genes contained the low temperature response element LTR, 31 *CtWRKY* genes contained the drought stress response element MBS, 35 *CtWRKY* genes contained the gibberellin response element GARE motif, and 28 *CtWRKY* genes contained defence and stress response element TC-rich repeats.

**Figure 5:**
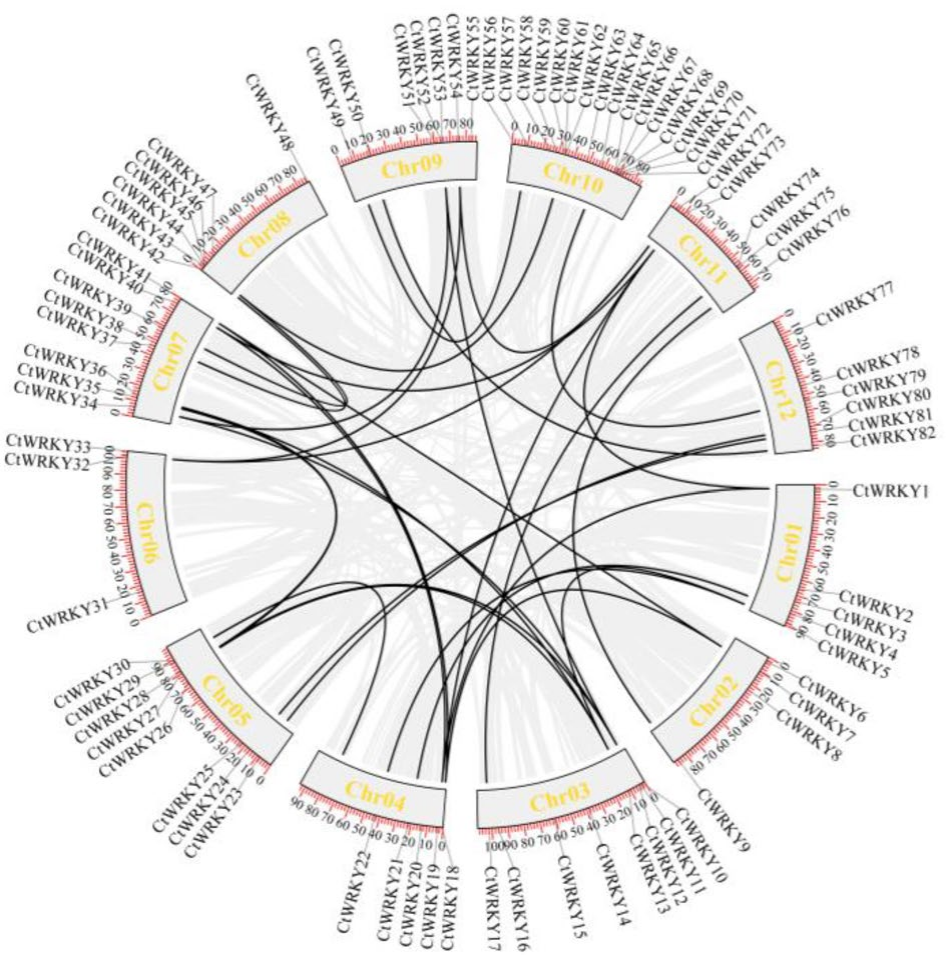
Collinear analysis of WRKY genes in safflower. The grey line represents all collinear regions in the entire safflower genome, and the black line represents the *CtWRKY* gene pair with replication events.

Fragment replication and tandem replication events are the main modes of gene family formation and expansion. The gene replication events of the safflower genome were analysed by the MCScanX tool (Fig. 5). The results revealed that 5 pairs of tandem duplicated gene pairs (*CtWRKY43/44*, *CtWRKY45/46*, *CtWRKY52/53*, *CtWRKY67/68* and *CtWRKY74/75*) were found in 82 *CtWRKY* genes, which were located on Chr8, Chr9, Chr10 and Chr11, respectively. Moreover, 34 pairs of fragment replication gene pairs were found. This indicates that fragment replication events may be the main driving force for the formation and expansion of the CtWRKY gene family. The Ka/Ks values of four CtWRKY gene pairs, which were NaN, were excluded. Furthermore, the Ka/Ks values of the remaining 34 gene pairs were below 0.5, and one gene pair exhibited a Ka/Ks value lower than 1, implying that these genes evolved via the process of purification selection. Tandemly duplicated genes had Ka/Ks values ranging from 0.27 to 0.62, whereas segmentally duplicated genes had Ka/Ks values ranging from 0.11 to 0.42. The mean Ka/Ks value for tandemly repeated genes (0.37) exceeded that of segmentally duplicated genes (0.22) (Table S3).

### Analysis of the expression pattern of the WRKY gene in safflower under different tissues and stress treatments

**Figure 6:**
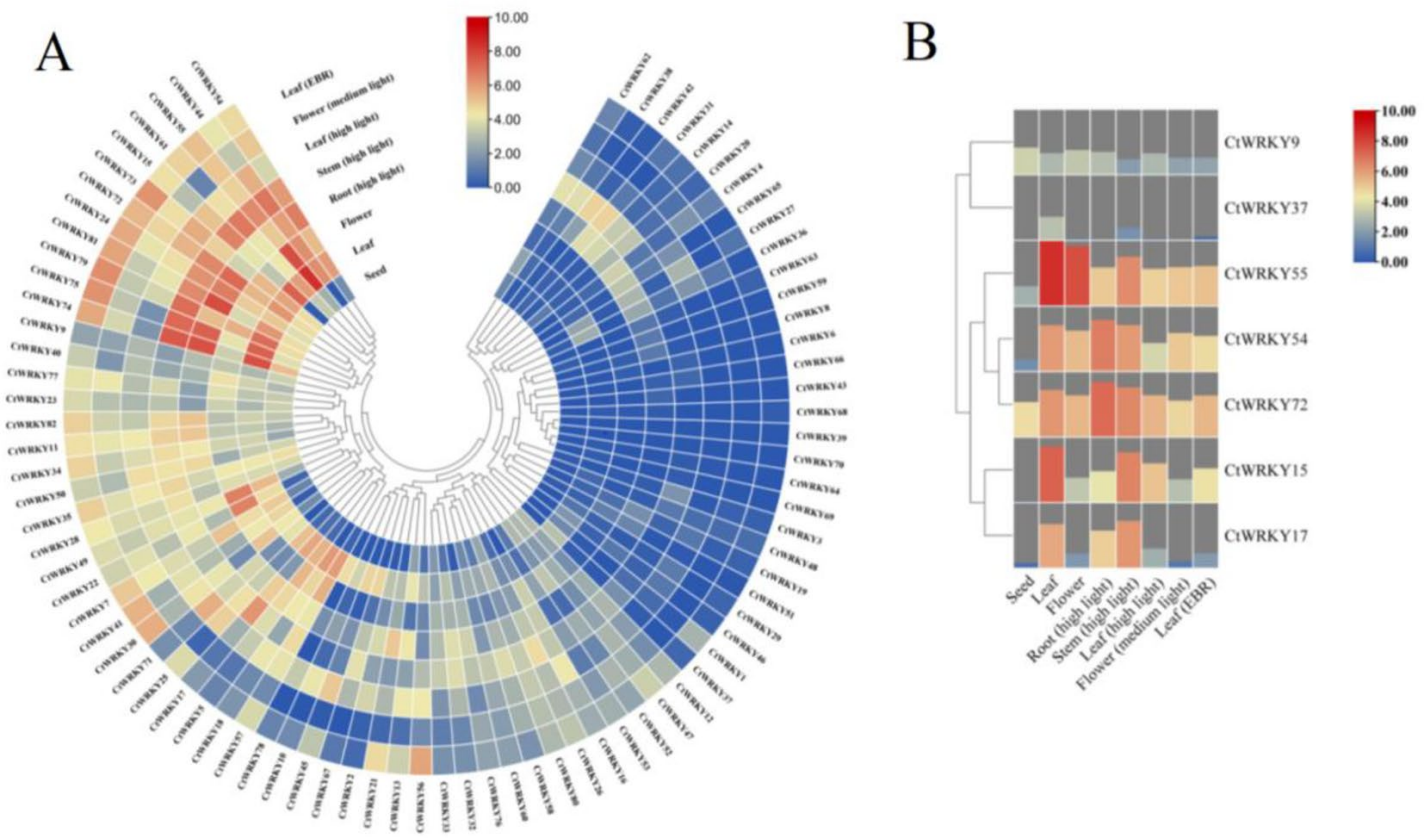
Expression of safflower WRKY genes in different tissues. (**A**) Expression heatmap of 82 *CtWRKY* genes in different tissues; (**B**) Expression heatmap of 7 *CtWRKY* genes with high homology to *Arabidopsis* drought-resistant WRKY genes in different tissues. The colours from blue to red represent different expression abundances. The light intensity was 40,000 lx (high light) and 20,000 lx (medium light), and the illumination time was 10 h/d. EBR treatment conditions: 0.1 μmol/L EBR solution was sprayed evenly on the front and back of the leaves.

To explore the potential function of the *CtWRKY* gene in plant growth and development, TPM values were calculated by TBtools software according to CtWRKY transcriptome data downloaded from the NCBI database (Table S4), and an expression heatmap of the *CtWRKY* gene in different tissues was generated (Fig. 6). The results showed that 82 *CtWRKY* genes were expressed in different tissues under the categories of seeds, leaves, flowers, EBR and light stress. However, the expression levels of a few genes were very low. Notably, 12 genes (*CtWRKY15*, − *24*, − *44*, − *54*, − *55*, − *61*, − *72*, − *73*, − *74*, − *75*, − *79*, and − *81*) showed high expression levels in 8 tissues, indicating that these genes play fundamental roles in growth and development and stress response. In addition, some *CtWRKY* genes were preferentially expressed in different tissues. For example, 4 *CtWRKY* genes (*CtWRKY11*, − *34*, − *35* and − *82*) were highly expressed in different tissues under EBR and light stress; 4 *CtWRKY* genes (*CtWRKY17*, − *22*, − *25* and − *49*) were preferentially expressed in leaves, flowers and roots; and only a few genes were expressed in seeds. Analysis of the expression levels of 7 CtWRKY genes (*CtWRKY9*, − *15*, − *17*, − *37*, − *54*, − *55*, and − *72*) with high homology to *Arabidopsis* drought tolerance genes in different tissues revealed that all 7 genes had high expression levels in leaves (Fig. 6). Additionally, 4 *CtWRKY* genes (*CtWRKY15*, − *54*, − *55*, and − *72*) showed high expression levels in flowers as well as in different tissues under EBG and light stress, suggesting that these *CtWRKY* genes may play an important role in abiotic stress.

### Analysis of the protein interaction network of the WRKY gene in safflower

**Figure 7:**
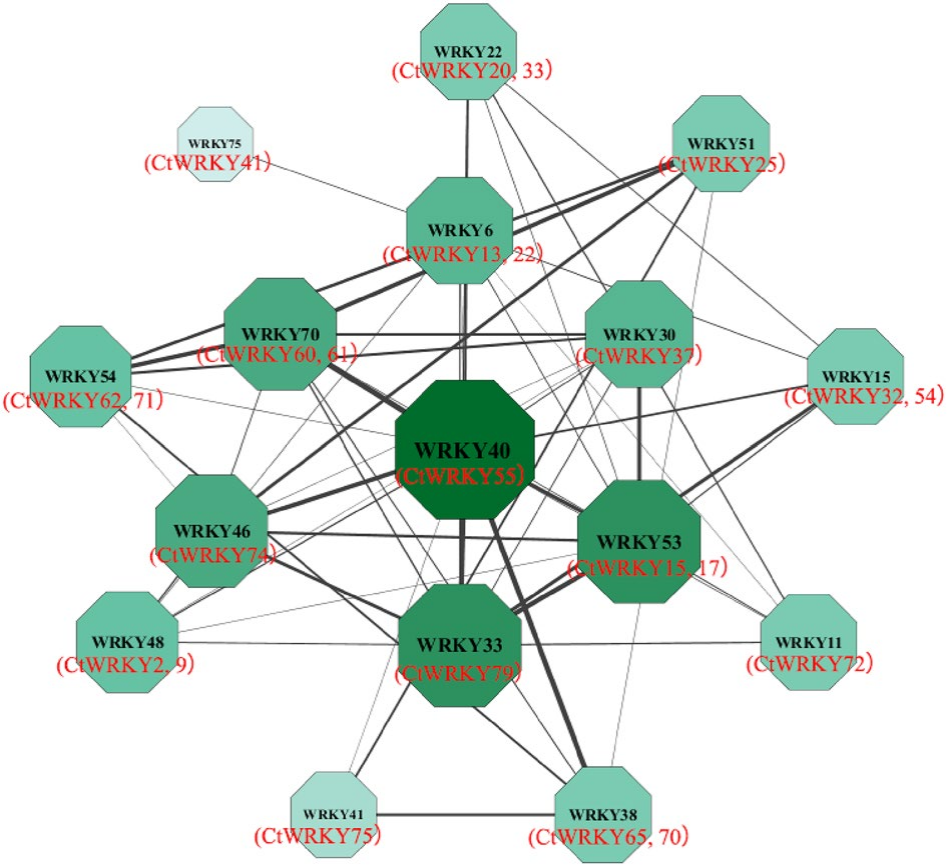
The protein interaction network of the WRKY protein from safflower. Cytoscape was used to visualize the network. The green coloured ball (node) represents the *CtWRKY* gene. The thickness of the connecting line represents the correlation between the two proteins.

To predict the potential regulatory role of CtWRKY proteins, a CtWRKY protein interaction network was constructed using the STRING database based on *Arabidopsis* WRKY protein interaction data (Fig. 7). The findings showed that the CtWRKY proteins with high sequence similarity to *AtWRKY30* (*CtWRKY37*), *AtWRKY33* (*CtWRKY79*), *AtWRKY40* (*CtWRKY55*) and *AtWRKY53* (*CtWRKY15/17*) were the hubs of the protein interaction network, and the remaining CtWRKY proteins had strong or weak interactions with *CtWRKY15*, *CtWRKY17*, *CtWRKY37*, *CtWRKY55* and *CtWRKY79*.

### Analysis of the expression pattern of the WRKY gene in safflower under different tissues and drought stress

**Figure 8:**
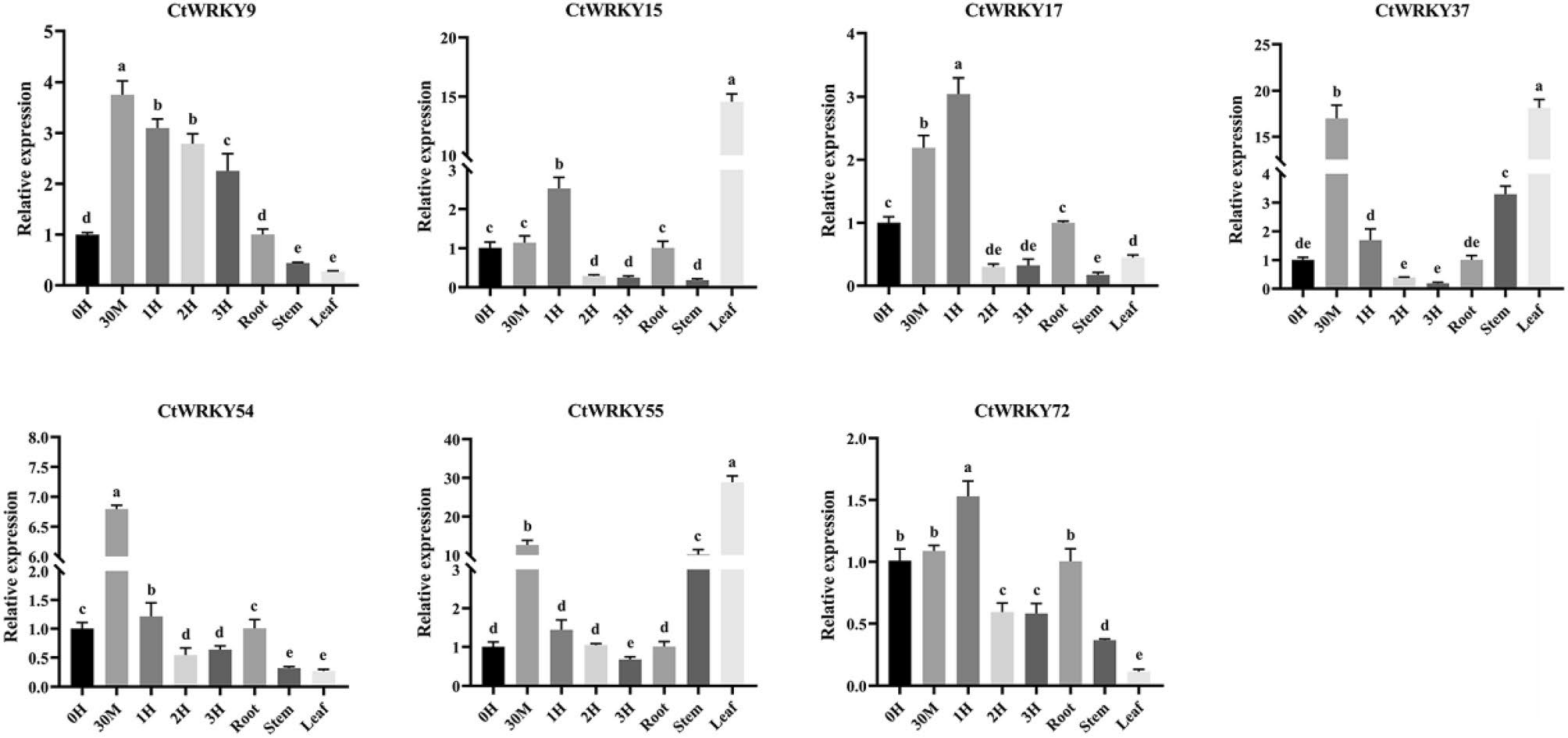
The relative expression of the WRKY gene in safflower under drought stress. qRT-PCR data were normalized using the safflower EF-1a and Actin genes. X-axes represent different treatments (0 h, normal conditions; 30 min, 1 h, 2 h, and 3 h indicate hours of drought treatment), and y-axes are scales of relative expression levels. Error bars are based on three biological replicates.

**Figure 9:**
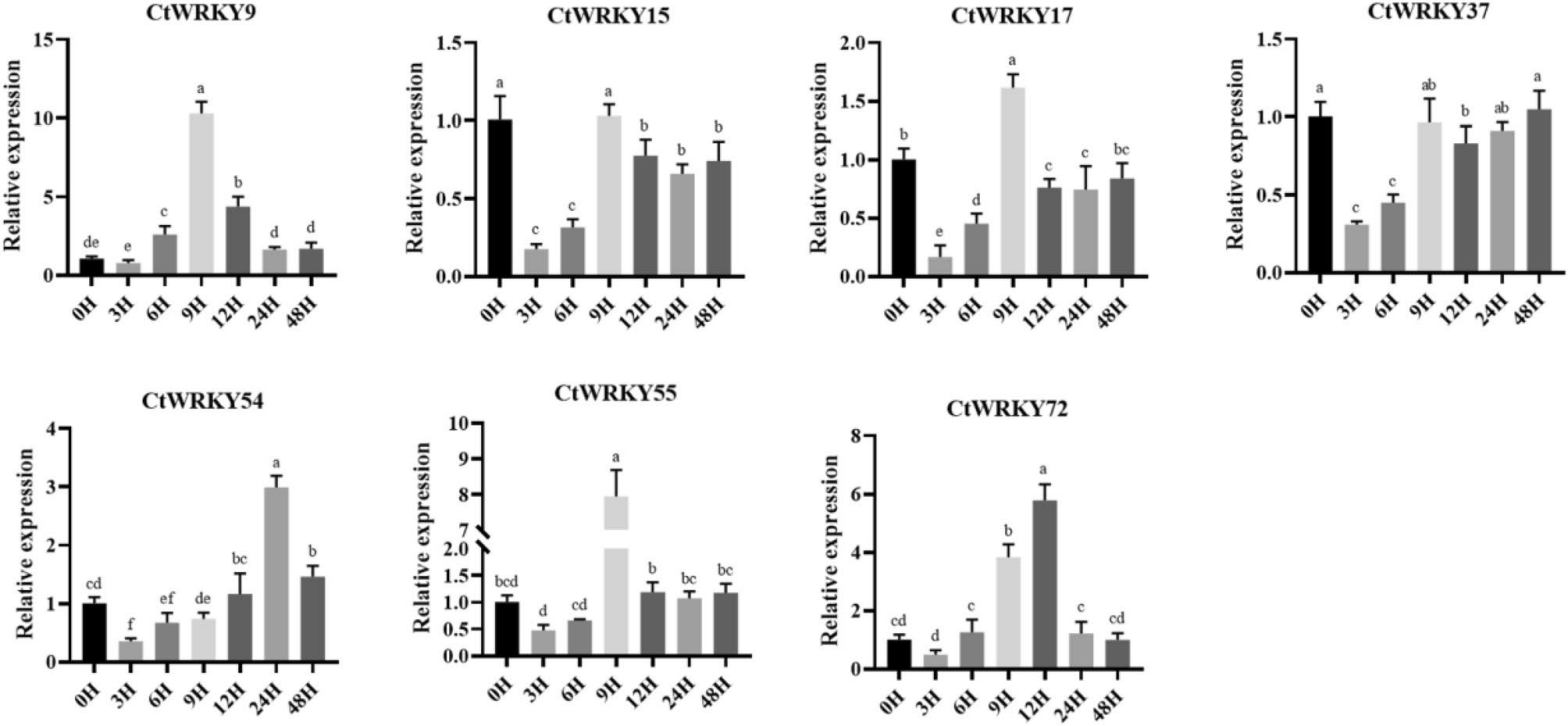
Relative expression level of the WRKY gene in safflower under drought-rewatering treatment. qRT-PCR data were normalized using the safflower EF-1a and Actin genes. X-axes represent various treatments (0 h, normal conditions; 3 h, 6 h, 9 h, 12 h, 24 h and 48 h indicate hours of drought-rewatering treatment), and y-axes are scales of relative expression levels. Error bars are based on three biological replicates.

Under adverse conditions, many stress-inducible genes can help plants resist the influence of adverse factors. To analyse the stress response of the *CtWRKY* gene under drought stress, seven *CtWRKY* genes with high homology to *Arabidopsis* drought tolerance genes were selected (group II: *CtWRKY9*, − *54*, − *55*, and − *72*; group III: *CtWRKY15*, − *17*, and − *37*), and their expression patterns in different tissues and under drought stress and drought-rewatering treatment were measured using qRT-PCR (Figs. 8 and 9). As shown in Figure S4, seven *CtWRKY* genes had a heptapeptide core sequence WRKYGQK and a zinc finger structure C_2_H_2_ or C_2_HC, which are very different from other drought tolerance genes except for the conserved domain. As shown in Fig. 8, seven *CtWRKY* genes were differentially expressed in roots, stems, and leaves. Three *CtWRKY* genes (*CtWRKY15*, − *37*, and − *55*) were highly expressed in leaves, and four *CtWRKY* genes (*CtWRKY9*, − *17*, − *54*, and − *72*) were highly expressed in roots, indicating that they play important roles in different tissues. The qRT-PCR analysis showed that the relative expression levels of the seven *CtWRKY* genes were significantly different within 3 h of drought treatment and 48 h of rewatering, and the overall expression pattern showed a trend of first increasing and then decreasing, indicating that these genes may play an important role in the drought stress response. As shown in Figs. 8 and 9, the expression levels of seven *CtWRKY* genes increased to varying degrees after drought stress and drought rehydration treatment, but the expression levels of these genes began to decrease as the treatment time increased. For example, after 30 min of drought treatment, three *CtWRKY* genes (*CtWRKY37*, − *54* and − *55*) showed high expression levels in leaf tissues (increased by > 6.5-fold), three *CtWRKY* genes (*CtWRKY9*, − *15* and − *17*) showed high expression levels in leaf tissues (increased by > 2.5-fold), and the expression level of *CtWRK72* increased 1.5-fold. After 9 h of rehydration, three *CtWRKY* genes (*CtWRKY9*, − *55* and − *72*) showed high expression levels (increased > 5.6-fold), and the expression level of *CtWRKY17* increased 1.6-fold. The expression level of CtWRKY54 increased 2.9-fold after 24 h of rehydration. In addition, the relative expression levels of the *CtWRKY37* and *CtWRKY55* genes rapidly increased more than 12-fold after drought treatment for 30 min, and the relative expression levels of the *CtWRKY9* and *CtWRKY55* genes rapidly increased more than eightfold after rehydration treatment for 9 h. This indicates that these genes may be involved more strongly or more rapidly in the response of plants to drought stress.

### Analysis of proline content, malondialdehyde content and antioxidant enzyme activity in safflower under drought stress

**Figure 10:**
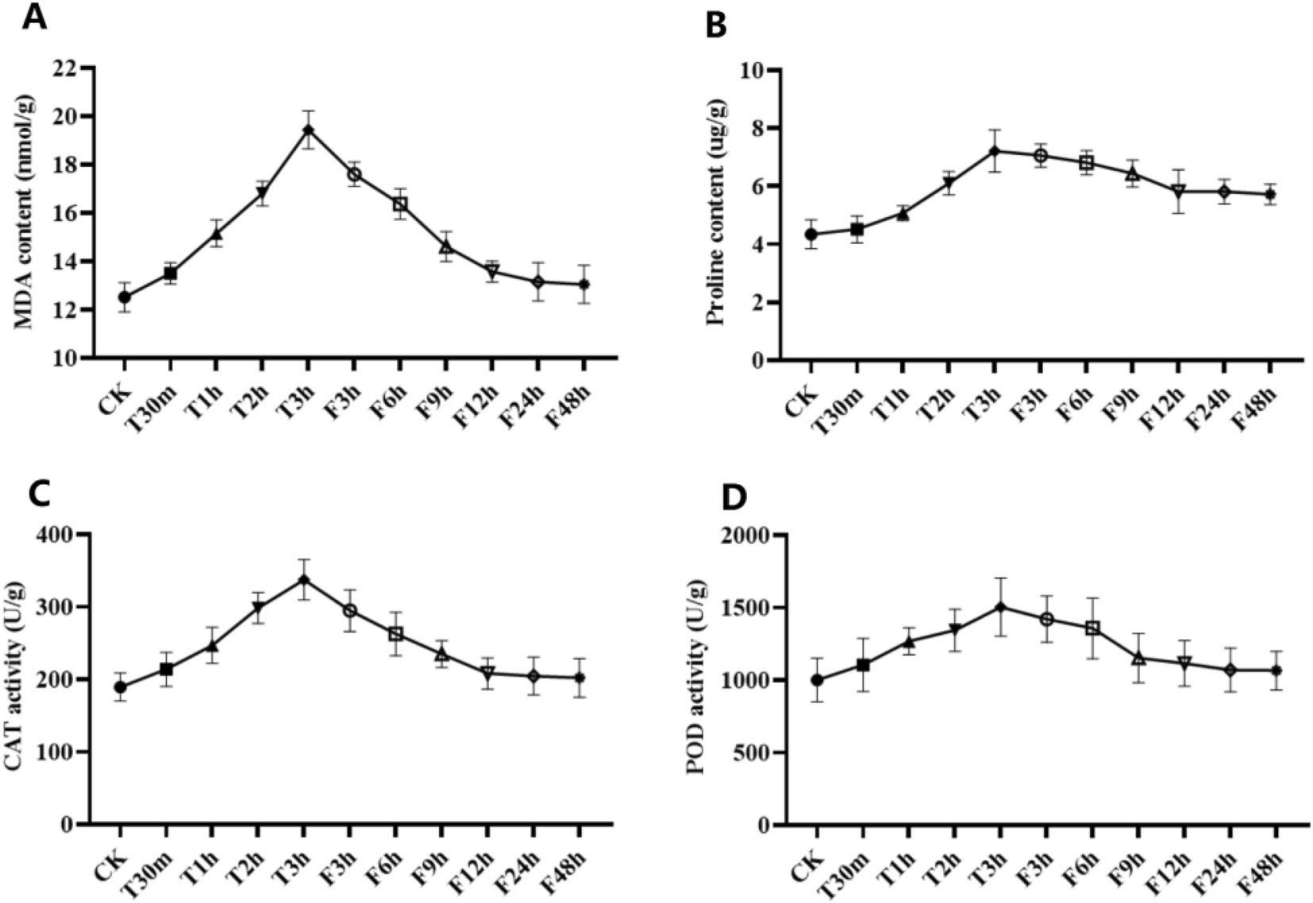
Effects of drought stress and rewatering on proline, malondialdehyde and antioxidant enzyme activities in safflower. (**A**) MDA: malondialdehyde content; (**B**) Pro: proline content; (**C**) CAT: catalase activity; (**D**) POD: superoxide dismutase activity. T30m, T1h, T2h and T3h represent drought stress time. F3h, F6h, F9h, F12h, F24h and F48h represent the rewatering time. Error bars are based on three biological replicates.

Osmotic stress occurs when plants have reduced water uptake ability under drought. To examine the stress response of saffron under drought, this study examined the proline content, malondialdehyde content, and antioxidant enzyme activities of safflower during drought stress and rehydration treatments (Fig. 10). As depicted in Fig. 10, the contents of Pro and MDA, as well as the activities of CAT and POD, exhibited an upwards trend during the 3-h period of drought stress, peaking at 3 h. However, as the rehydration time increased, there was a subsequent downwards trend in these variables, resulting in an overall increasing and then decreasing pattern. Both MDA and Pro contents reached their highest point after 3 h of drought stress, with respective increases of 1.55- and 1.66-fold compared to the control (Fig. 10A,B). After rehydration, both contents began to decrease and stabilized after 12 h but did not return to the control level. During drought stress, CAT and POD activities continued to increase and reached their peak at 3 h, showing increases of 1.78- and 1.51-fold, respectively, when compared with the control (Fig. 10C,D). After rehydration, their activities began to decline at 12 h and then stabilized, although they remained slightly higher than the control level. These findings suggest that safflower maintained a balance between the production and elimination of ROS during drought stress and rehydration.

## Discussion

The WRKY transcription factor family is a plant-specific supergene family that plays a key role in the growth and development of plants as well as biotic and abiotic stresses. Currently, 64, 54, 138, 102, and 125 members of the WRKY gene family have been identified in strawberry, shantung maple, mandarin orange, flax and maize^1,4,5,8,28^. In this study, a total of 82 *CtWRKY* genes were identified from the safflower genome data, which were unevenly distributed on 12 chromosomes (Fig. 2)^28^. Furthermore, *CtWRKY43*, − *44*, − *45*, and − *46* were grouped together in the identical chromosomal segment, creating a gene cluster, which indicates that these genes might share common biological roles since they are regulated and expressed closely together^5^. The 82 CtWRKY genes can be classified into three major groups and five subgroups, which is consistent with the results of previous studies (Fig. 1 and Fig. S1)^3,8^. Group II contains 42 WRKY proteins, which can be further divided into five subgroups according to the variation in protein sequence, suggesting that group II may be the main driving force in the expansion of the WRKY gene family^4–6,8^. Among them, group IIa has only two WRKY proteins, which is probably due to its shorter evolutionary history compared to the other subgroups^5,29^. Furthermore, the WRKY domain at the C-terminus of Group I members was found to contain the same conserved motif as the WRKY domain of group II and group III members, supporting the hypothesis that mutation or deletion of the C-terminal domain of Group I may have been the cause of the evolution of the WRKY domain of Group II and Group III members^5,30,30,31^.

Multiple sequence alignment revealed that most CtWRKY proteins have a highly conserved heptapeptide core sequence (WRKYGQK) and zinc finger structure (C_2_H_2_ or C_2_HC)^28^. Moreover, three types of heptapeptide sequence variations (WKKYGEK, WRKYGKK and WRKYGHK) were found, indicating that the variation in the domain may be one of the reasons for the expansion of *WRKY* genes in safflower. This phenomenon has been observed in the *WRKY* members of strawberry, *Isatis* indigotica, rice, and apple^28,32–34^. Reportedly, the WRKY domain of *GmWRKY6* and *GmWRKY21* is a heptapeptide variant sequence WRKYGKK, which has lost the ability to bind to the W-box, affecting the expression pattern of stress-responsive genes of WRKY transcription factor targets^35^. It is speculated that these variations may change the binding specificity of DNA targets and influence the expression status of stress-responsive genes targeted by CtWRKY transcription factors.

Analysis of gene structure and conserved motifs can provide essential information for understanding the evolution of gene families^36^. As shown in Fig. 3, the conserved motifs and exon/intron distribution types of *CtWRKY* genes in the same group or subgroup have good similarity, indicating that the members of the same group are highly conserved and functionally similar during the evolutionary process, which is very common in plants^5,37,38^. In addition, 10 conserved motifs (length: 6–30 amino acid residues) were identified in 82 *CtWRKY* genes (Fig. S2 and Table S2). Gene replication events (fragment replication or tandem replication) are thought to be the key force in amplifying gene families in angiosperm evolution^38^. As shown in Fig. 5 and Table S3, 5 tandem duplicated gene pairs and 34 fragment repeated gene pairs were found in CtWRKY TFs, involving 68 *CtWRKY* genes, accounting for 82.93% of the members of the safflower WRKY family and indicating that fragment duplication events play a crucial role in the expansion of the CtWRKY gene family. Normally, a ratio of 1 indicates neutral selection; a Ka/Ks ratio > 1 means adaptive evolution with positive selection, while a ratio < 1 means negative or purifying selection^40,41^. As a result, all the Ka/Ks ratios for the 34 gene pairs were < 1 (Table S3), indicating that the CtWRKY gene family is highly conserved during evolution, consistent with previous research^5^.

Based on the interaction analysis of WRKY proteins in other species, the potential regulation of CtWRKY proteins, gene function and its relationship with the overall biological function can be predicted. In this study, the STRING database was used to construct a CtWRKY protein interaction network using *Arabidopsis thaliana* as a template. As illustrated in Fig. 7, four CtWRKY proteins (*CtWRKY15*, *CtWRKY17*, *CtWRKY55* and *CtWRKY79*) exhibited high sequence similarity with *AtWRKY33*, *AtWRKY40* and *AtWRKY55* and were identified as central nodes within the interaction network. These proteins displayed varying degrees of interaction with others. Reportedly, *AtWRKY40* can form dimers with *AtWRKY18* or *AtWRKY60* to modify pathogen resistance, regulate the osmotic and antioxidant capacity of plants, and improve the drought tolerance of plants^42–44^. *AtWRKY33* can enhance *Arabidopsis* resistance to fungal pathogens and improve *Arabidopsis* resistance to low temperature and high salt stress^45–47^. *AtWRKY53* can regulate stomatal movement by reducing hydrogen peroxide content or promoting starch degradation, thereby regulating drought tolerance in *Arabidopsis*^48,49^. It is widely accepted that homologous proteins with similar domains and sequences across different species may possess identical or similar functions^5^. Hence, it is conjectured that *CtWRKY15*, *CtWRKY17*, *CtWRKY55*, and *CtWRKY79* might participate in a transcriptional regulatory mechanism, along with others, in the response of safflower to drought stress. In summary, various strong or weak interactions exist among CtWRKY proteins, indicating potential coexpression in response to biotic and abiotic stresses, with consequent regulation of safflower stress resistance.

In this study, we analysed transcriptome data for safflower retrieved from the NCBI database. The results showed that 82 *CtWRKY* genes had tissue-specific expression patterns under normal and stress treatments (Fig. 6), which was consistent with the results in other plants, such as *Acer truncatum*, *Linum usitatissimum* L., *Platycodon grandiflorum* and *Zea mays*^1,5,8,50–53^. However, the expression levels of some of these genes were low, suggesting that these genes may be pseudogenes or may have specific spatial and temporal expression patterns that were not investigated in this database^5,8^. As shown in Fig. 6B, 7 *CtWRKY* genes (*CtWRKY9*, − *15*, − *17*, − *37*, − *54*, − *55*, and − *72*) were highly expressed in leaves. Moreover, 4 *CtWRKY* genes (*CtWRKY15*, − *54*, − *55*, and − *72*) had high expression levels in different tissues and were induced by EBG and light stress, indicating that these genes may play an important role in specific tissues and abiotic stresses^8,28^. For instance, *ZmWRKY79-*mediated drought tolerance positively depends on an increase of ABA levels by triggering ABA biosynthetic genes including *AAO3* genes^13^. *AtWRKY57* stimulates the expression of *RD29A* and *NCED3* by attaching to its W-box element, thereby boosting drought tolerance in *Arabidopsis*^54^. *TaWRKY51* is a positive regulator of root architecture and grain yield-contributing traits^55^. These findings indicate that WRKY transcription factors display diverse expression profiles in different organs or tissues, regulating a range of biological and physiological metabolic processes in safflower^1,3,8^.The cis-acting element is an important “switch” used to regulate gene expression in plants. Promoter analysis has identified numerous abiotic stress response cis-elements in the promoter regions of *WRKY* genes across multiple species, indicating that most plant *WRKY* genes may play a role in controlling transcriptional responses to abiotic stress^25,26^. In this study, a variety of cis-acting elements, such as light-responsive elements, abiotic stress-responsive elements, and hormone-responsive elements, were identified in the promoter region of the *CtWRKY* gene, which is important for the study of the potential function of the *CtWRKY* gene and is in agreement with the results of previous studies (Fig. 4 and Fig. S3). Previous research has demonstrated that WRKY transcription factors participate in the response to abiotic stress via stress-related transcriptional regulatory elements. For example, various environmental factors, including drought, salinity, and high temperature, alongside defence-related plant hormones such as SA and ABA, can trigger the gene transcription of *ZmWRKY65*, thereby increasing its tolerance to abiotic stress^56^. The expression of the *McWRKY57* gene was stimulated by mannitol, ABA, and MeJA. Overexpression of *McWRKY57-like* in *Arabidopsis* plants considerably improved plant drought tolerance^57^. Additionally, transgenic *Arabidopsis* lines overexpressing of *EjWRKY17* exhibited increased cotyledon greening and root elongation under abscisic acid (ABA) treatment^58^. In summary, the research findings offer new insights for safflower in uncovering the signalling pathways in response to abiotic stress.

WRKY transcription factors are crucial in plant responses to drought, high temperatures, and salt stress^6,7,50,51^. It has been reported that *SbWRKY30* is expressed predominantly in sorghum leaves and roots. Overexpression of this gene in *Arabidopsis* has been shown to enhance drought tolerance, likely through interference with root structure and induced expression of the *AtRD19* gene^12^. The *GhWRKY91-*transformed plants displayed enhanced drought tolerance and delayed senescence of leaves under drought conditions. This was associated with increased expression of stress-responsive genes and decreased expression of senescence-associated genes (*SAGs*)^59^. To study the stress response of the *CtWRKY* gene under drought stress, seven *CtWRKY* genes with high homology to *Arabidopsis* drought tolerance genes were selected (group II: *CtWRKY9*, − *54*, − *55*, and − *72*; group III: *CtWRKY15*, − *17*, and − *37*). qRT-PCR was used to analyse their expression patterns in different tissues and under drought stress and drought rewatering treatments. As shown in Figs. 8 and 9, the relative expression levels of these *CtWRKY* genes were differentially upregulated from 0 to 3 h under drought stress, showing a trend of increasing and then decreasing, suggesting that these genes may be crucial at the early stage of drought stress treatment and have a positive or negative regulatory effect on drought stress; however, their regulation varies with the degree of stress. Under drought-rehydration treatment, the expression levels of *CtWRKY* genes were significantly upregulated after 9 h. However, following rehydration, the downregulation of *CtWRKY* genes was relatively minor. These results suggest that these genes may play a role in the plant late-stage recovery from drought stress. This difference in the levels of expression before and after drought stress indicates that WRKY transcription factors respond to drought stress in a time-sensitive manner, consistent with previous studies^60,61^. The expression level of *ZmWRKY40* can peak rapidly at 10 h after drought treatment, and its overexpression can improve drought tolerance in transgenic *Arabidopsis* through the regulation of stress-related genes (*DREB2B* and *RD29A*)^62^. In addition, the expression levels of *CtWRKY9* and *CtWRKY55* increased rapidly under drought and rehydration treatments compared to the control, reaching a peak of more than eightfold, implying a significant role in the response to drought stress. Such observations suggest that these genes should be explored further to understand their potential as key regulators under drought conditions. However, the underlying mechanism of this phenomenon remains to be further elucidated. Combined with the results of functional studies of the WRKY family in many plants, it can be speculated that more *CtWRKY* genes have regulatory functions related to abiotic stress that are waiting to be discovered.

Drought stress exposes plants to osmotic stress, which leads to an increase in ROS production, resulting in peroxidation of plant cell membranes. Plants can actively accumulate osmotic solutes or antioxidant enzymes to reduce the damage caused by drought^63,64^. Wei et al. reported higher antioxidant enzyme activity in drought-tolerant safflower varieties under drought stress^65^. In this study, the Pro content, MDA content, CAT activity and POD activity of safflower under drought stress and rehydration treatment were determined. As shown in Fig. 10, the contents of Pro, MDA, CAT and POD in safflower under drought stress and rehydration treatments showed a trend of first increasing and then decreasing, which was similar to the findings in oak^66^. However, after rewatering, the MDA level did not decrease to the level of the control sample, indicating that drought stress can cause irreversible damage to plants. Furthermore, the levels of CAT and POD were still slightly higher than those in the control after 48 h, indicating that drought stress may cause damage to the antioxidant oxidase system. Çulha Erdal et al. found that drought stress had a negative effect on proline content, MDA content and antioxidant enzyme activity in safflower, which was difficult to fully recover even after rewatering^67^. Notably, the expression level of the *CtWRKY* gene was high after drought stress. In addition, the expression level of genes was also significantly upregulated after rewatering, showing a trend of first increasing and then decreasing. In conclusion, it is speculated that the *CtWRKY* gene may regulate the ROS level of plants and enhance the drought resistance of plants.

## Conclusion

Safflower is an important economic crop with medicinal and food utility. In this study, 82 CtWRKY transcription factors were identified from the safflower genome, and bioinformatics and gene expression pattern analysis were performed to reveal the important functions of *CtWRKY* genes in plant growth and development and abiotic stress response under drought treatment. The qRT-PCR analysis showed that seven *CtWRKY* genes were responsive to drought stress treatment, indicating that they had positive or negative regulatory effects on drought stress. In addition, *CtWRKY9* and *CtWRKY55* were found to be more involved or more rapidly involved in the drought stress response process, providing a basis for the future mining of candidate genes related to drought tolerance in safflower. The results provide a theoretical basis for further exploring the function of the *CtWRKY* gene in safflower growth and development and response to abiotic stress.

## Materials and methods

### Genome-wide identification and physicochemical analysis of the safflower WRKY gene family

Our study complies with relevant institutional, national, and international guidelines and legislation. The whole-genome sequence, CDS, protein sequence and annotation files for safflower were provided by the Institute of Economic Crops, Xinjiang Academy of Agricultural Sciences. Identification of the safflower WRKY gene family was performed using two methods: 1. The protein sequence of the AtWRKY gene family was downloaded from the NCBI database (https://www.ncbi.nlm.nih.gov/). The protein sequence of the AtWRKY gene family was used as a template, and the protein sequence of the safflower WRKY gene family was preliminarily screened by the Basic Local Alignment Search Tool (BLAST) of TBtools software^1^, 2. The hidden Markov model (HMM) file of the WRKY domain (PF03106), downloaded from the Pfam protein family database (http://pfam.sanger.ac.uk/), was used for the identification of WRKY genes from the safflower genomic database by TBtools^5,8,68^. Subsequently, the online tools Pfam (http://pfam.xfam.org/search#tabview=Table 1), SMART (http://smart.embl.de/) and NCBI CDD (https://www.ncbi.nlm.nih.gov/cdd/) were used to verify whether the CtWRKY protein had a conserved WRKY domain, and the candidate protein sequences without conserved domains were removed^8^. The ExPASy website (https://www.ExPASy.org/) was used to predict the physical and chemical properties of CtWRKY proteins, including molecular weight, isoelectric point and hydrophilicity, and the parameters were set as default values^69^. The online tool WoLF PSORT (https://wolfpsort.hgc.jp/) was used to predict the subcellular localization of CtWRKY family members^70^.

### Phylogenetic analysis and chromosome mapping of the safflower WRKY gene family

The whole genome, protein sequence, annotation file, and protein sequence of the WRKY gene family were downloaded from The *Arabidopsis* Information Resource (TAIR) database (https://www.arabidopsis.org/)^71^. DNAMAN 9.0 software was used for multiple sequence alignment of AtWRKY gene family proteins in safflower and *Arabidopsis*. The phylogenetic tree was constructed by the neighbour-joining (NJ) method using MEGA 7.0 software, a with 1000 bootstrap replicates^72^. The chromosome location map of the CtWRKY gene family was drawn by TBtools software according to the gene annotation file of the safflower genome.

### Gene structure, promoter and collinearity analysis of the safflower WRKY gene family

The intron and exon characteristics of the *CtWRKY* gene were analysed by TBtools software according to the annotation information of the *CtWRKY* gene^73^. The conserved motifs of the CtWRKY protein were identified using the MEME online tool (http://meme.sdsc.edu/meme/)^74^. The following parameters were used: any number of repetitions, maximum of 10 motifs, and an optimum motif width of 6 to 150 amino acid residues. The gene structure and conserved motif results were visualized using TBtoolssoftware.

The sequence of the promoter region 2000 bp upstream of the CDS region of the *CtWRKY* gene was extracted using TBtools software, and the online tool PlantCARE (http://bioinformatics.psb.ugent.be/webtools/plantcare/html/) predicted the cis-acting elements of the *CtWRKY* gene and analysed the stress and hormone response elements^1,5,8,75^. Collinearity data of *CtWRKY* genes were obtained using the MCScanX tool, and the fragment replication and tandem replication genes were identified according to the safflower genome data^76^. Determination of gene duplication events was performed according to the following criterion: If two genes were located on the same chromosomal region and were adjacent to each other or separated by one gene, they were considered tandemly duplicated genes^39,77^. Based on the CtWRKY protein and CDSs, the Ka, Ks and Ka/Ks values of the Ct*WRKY* gene were calculated using the Simple Ka/Ks Calculator of TBtools software.

### Transcriptome analysis of the safflower WRKY gene family

The RNA-seq data for safflower under the categories of seed, leaf, flower, epibrassinolide (EBR) and light stress were downloaded from the NCBI-SRA database (Biological project: PRJNA76135; PRJNA628030; PRJNA493984)^91^. The light intensity was 40,000 lx (high light) and 20,000 lx (medium light), and the illumination time was 10 h/d. EBR treatment conditions: 0.1 μmol/L EBR solution was sprayed evenly on the front and back of the leaves. The expression patterns of *CtWRKY* gene in different tissues such as roots, stems, leaves and flowers were calculated by TBtools software. The expression values were calculated by log2 (TPM: transcripts per million) and were displayed as a heatmap by TBtools software^68^.

### Protein interaction network analysis of the safflower WRKY gene family

The CtWRKY protein‒protein interaction (PPI) network was constructed using the Search Tool for the Retrieval of Interacting Genes/Proteins (STRING) database (https://string-db.org/) with the *Arabidopsis* WRKY protein as a template^5,78^. According to the protein‒protein interaction score (≥ 0.400), the number of interactions was 5. The thicker the line between the targets in the interaction network, the stronger the interaction. The results were imported into Cytoscape software to visualize the protein‒protein interaction network^79^.

### Plant materials, drought stress treatment and tissue collection

The safflower seeds (21TY012) used in this experiment were provided by the Institute of Economic Crops, Xinjiang Academy of Agricultural Sciences. Safflower seeds were planted in pots and cultivated under ambient temperature of 25 °C and 16 h/8 h (light/dark) light conditions for 14 days. Safflower seedlings with uniform growth were selected, and their roots were soaked in 1/2 Hoagland nutrient solution for 9 days. The roots were then soaked in 11% PEG-6000 solution to simulate drought stress. With 0 h treatment as a control, seedling samples were collected at 0 h, 30 min, 1 h, 2 h and 3 h after drought treatment and 3 h, 6 h, 9 h, 12 h, 24 h and 48 h after rehydration. Roots, stems, and leaves were immediately frozen in liquid nitrogen and stored at − 80 °C until RNA extraction.

### Analysis of safflower WRKY gene expression under drought stress by qRT-PCR

The RNAprep Pure plant kit (TIANGEN, China) was used to extract total RNA from different treated seedling samples according to the manufacturer’s instructions, and the concentration and quality of RNA were determined by spectrophotometry. Then, the RNAs were reverse transcribed into first-strand cDNA by a FastKing RT Kit (with gDNase) (TIANGEN, China) and stored at − 20 °C.

Seven *CtWRKY* genes (*CtWRKY9*, − *15*, − *17*, − *37*, − *54*, − *55 and −* *72*) with high homology to *Arabidopsis* drought tolerance genes *AtWRKY11*, − *17*,* −* *30*, − *40*, − *46*, − *53 and −* *57* were selected for qRT-PCR analysis^11,42,43,48,54,80,81^. The reference genes were *EF-1a* (Accession No.: KJ634806) and *Actin* (Accession No.: KJ634809). Gene-specific primers were designed using Primer 5.0 software, and the primer sequences are shown in Table S5. DNAMAN 9.0 software was used to perform multiple sequence alignment of 9 candidate CtWRKY proteins with drought-resistant WRKY proteins from *Arabidopsis thaliana*, *Chrysanthemum morifolium* Ramat, *Helianthus annuus* L. and *Triticum aestivum* L.^59,62,82–90^.

Quantitative real-time PCR was performed using a CFX96 TouchTM Real-Time PCR detection system (Bio-Rad, USA). The qRT-PCR amplification program and reaction system configuration were performed according to the manufacturer’s instructions for SuperReal PreMix Plus (SYBR Green) (TIANGEN, China). The quantitative data were calculated using the 2^−∆∆Ct^ method^92^. The amplification conditions were as follows: initial denaturation at 95 °C for 15 min and 40 cycles of denaturation at 95 °C for 10 s, annealing at 50–60 °C for 20 s and extension at 72 °C for 20 s. Finally, a melting curve was used to check the amplification specificity.

### Determination of antioxidant enzyme activity and proline and malondialdehyde (MDA) contents

The CAT activity was determined by ammonium molybdate colorimetry (Boxbio, AKAO003 Assay Kit, China). The POD activity was determined by the guaiacol method (Boxbio, AKAO005C Assay Kit, China). The MDA content was determined by thiobarbituric acid colorimetry (Boxbio, AKFA013C Assay Kit, China). The Pro content was measured using the ninhydrin colorimetric method (Boxbio, AKAM003C Assay Kit, China).

### Supplementary Information

Supplementary Information.

## Data Availability

RNA-seq fastq files were deposited in NCBI Sequence Read Archive (SRA) under accession number PRJNA76135, PRJNA628030 and PRJNA493984. Genome, proteome, and annotation files were available in *Carthamus tinctorius* database (https://safflower.scuec.edu.cn/).
