# Supplementary material for: Genome-wide identification and comprehensive analysis of WRKY transcription factor family in safflower during drought stress
Source: Sci Rep. 2023 Oct 7;13:16955. doi: 10.1038/s41598-023-44340-y (PMC10560227; doi:10.1038/s41598-023-44340-y)
Supplement: Supplementary file 1 — Supplementary Information. [file 41598_2023_44340_MOESM1_ESM.doc]

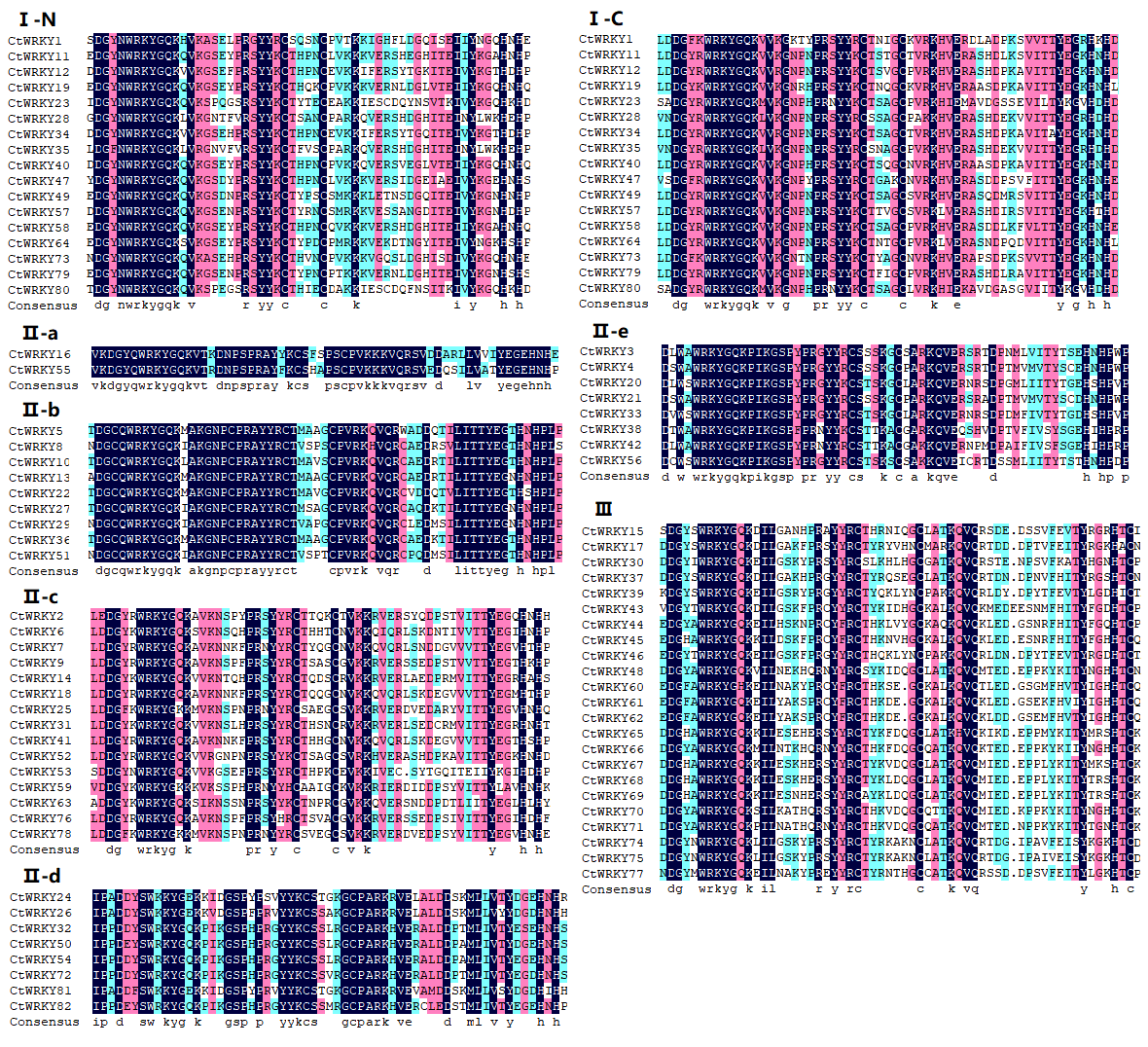


Figure S1. Multiple sequence alignment of the WRKY domains in safflower. Alignment using DNAMAN software.

**
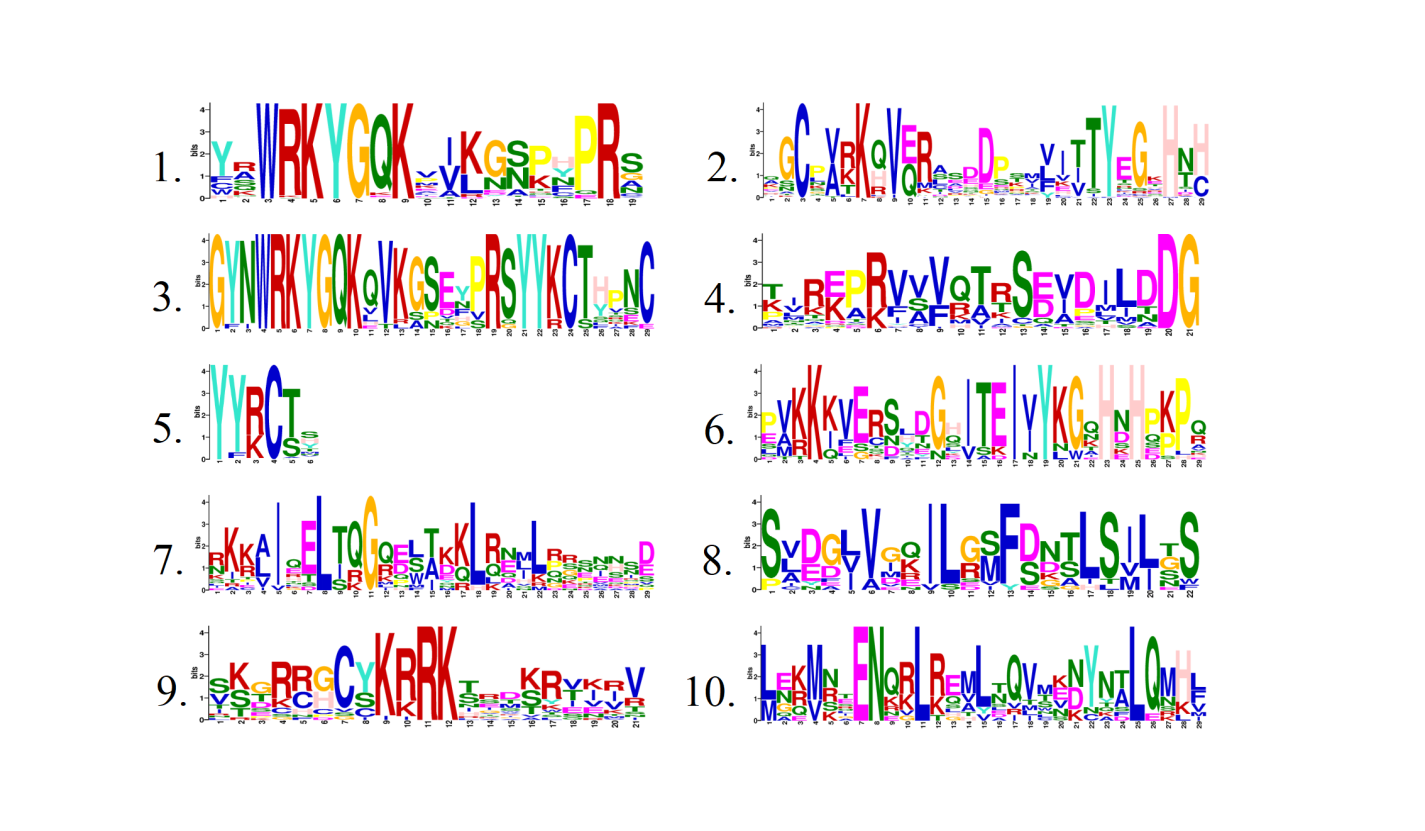
**

Figure S2. Conserved motifs of CtWRKY protein sequences. The Y axis (measured in bits) depicts the overall height of the stack, indicating the sequence conservation at that position, while the height of symbols within the stack indicates the relative frequency of each amino at that position.

Figure S3. The number of WRKY genes containing various cis-acting elements.


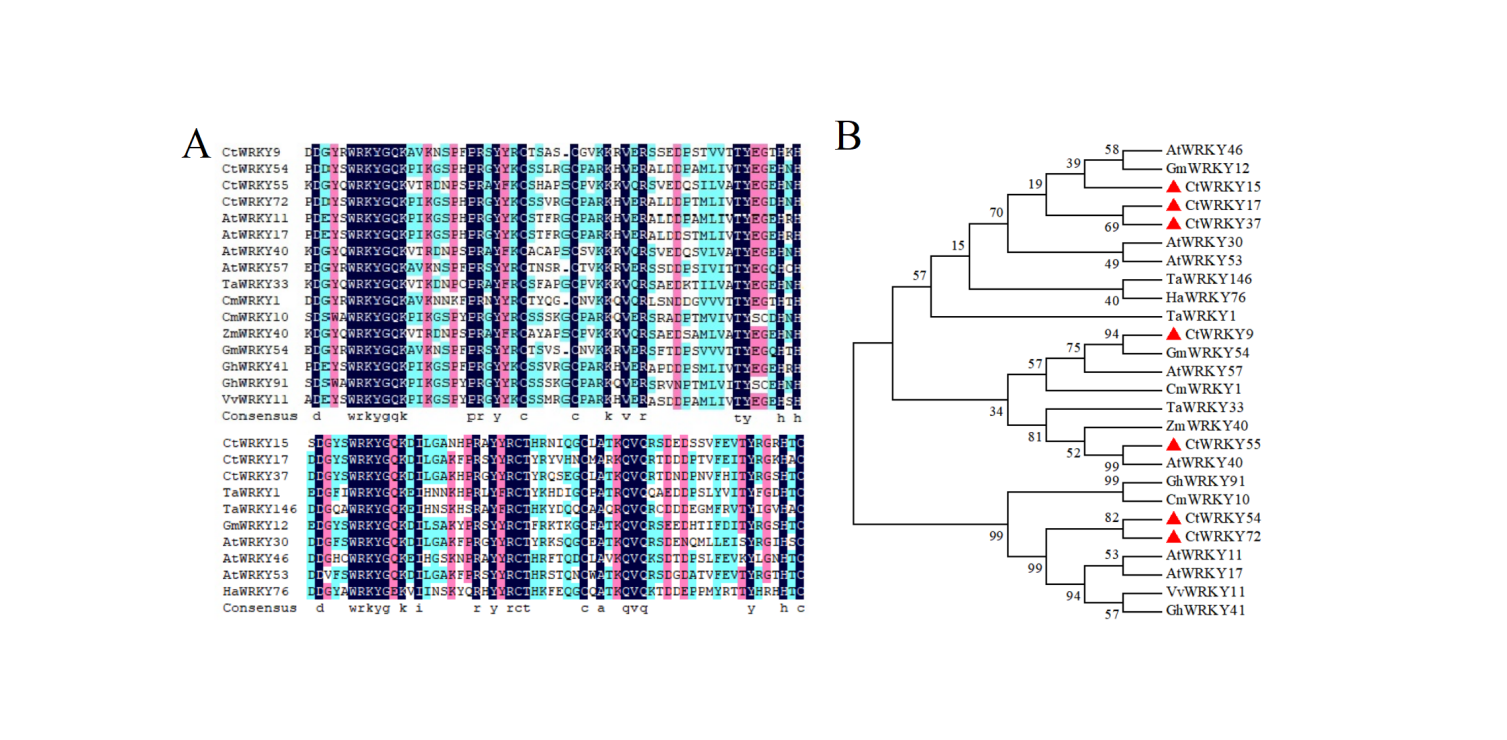


Figure S4. Multiple alignment and phylogenetic relationship between CtWRKY gene and drought-resistant WRKY genes in Arabidopsis thaliana, sunflower and chrysanthemum. **A**: Multiple alignment of CtWRKY protein with drought-resistant WRKY gene protein sequences of Arabidopsis thaliana, sunflower and chrysanthemum. **B**: Phylogenetic relationship between CtWRKY genes and WRKY genes in other crops.

Table S1. Analysis of the physicochemical properties of WRKY gene family members in safflower

| Gene ID | Gene name | Chr | Length  (aa) | CDS Length(bp) | MW（Da) | PI | Gravy | Subcellular Localization |
| --- | --- | --- | --- | --- | --- | --- | --- | --- |
| evm.model.Hic_asm_10.184 | CtWRKY1 | Chr01 | 337 | 1014 | 37967.73 | 8.40 | -0.751 | Nucleus |
| evm.model.Hic_asm_10.3106 | CtWRKY2 | Chr01 | 329 | 990 | 36472.18 | 6.21 | -0.922 | Nucleus |
| evm.model.Hic_asm_10.3426 | CtWRKY3 | Chr01 | 427 | 1284 | 46793.35 | 5.77 | -0.818 | Nucleus |
| evm.model.Hic_asm_10.3862 | CtWRKY4 | Chr01 | 295 | 888 | 33161.24 | 5.07 | -1.043 | Nucleus |
| evm.model.Hic_asm_10.4252 | CtWRKY5 | Chr01 | 547 | 1644 | 59196.68 | 6.31 | -0.648 | Nucleus |
| evm.model.Hic_asm_0.225 | CtWRKY6 | Chr02 | 226 | 681 | 25763.81 | 8.37 | -0.881 | Peroxisome |
| evm.model.Hic_asm_0.819 | CtWRKY7 | Chr02 | 196 | 591 | 22438.86 | 8.40 | -1.032 | Nucleus |
| evm.model.Hic_asm_0.1680 | CtWRKY8 | Chr02 | 482 | 1449 | 52758.86 | 7.98 | -0.655 | Peroxisome |
| evm.model.Hic_asm_0.4048 | CtWRKY9 | Chr02 | 327 | 984 | 35872.15 | 5.53 | -0.937 | Nucleus |
| evm.model.Hic_asm_9.64 | CtWRKY10 | Chr03 | 451 | 1356 | 49935.83 | 7.31 | -0.646 | Nucleus |
| evm.model.Hic_asm_9.128 | CtWRKY11 | Chr03 | 700 | 2103 | 76330.96 | 6.01 | -0.889 | Nucleus |
| evm.model.Hic_asm_9.214 | CtWRKY12 | Chr03 | 540 | 1623 | 59145.18 | 6.36 | -0.796 | Nucleus |
| evm.model.Hic_asm_9.680 | CtWRKY13 | Chr03 | 598 | 1797 | 64203.75 | 6.05 | -0.673 | Nucleus |
| evm.model.Hic_asm_9.2026 | CtWRKY14 | Chr03 | 204 | 615 | 23582.62 | 9.00 | -0.799 | Nucleus |
| evm.model.Hic_asm_9.2879 | CtWRKY15 | Chr03 | 308 | 927 | 35193.38 | 5.66 | -0.786 | Nucleus |
| evm.model.Hic_asm_9.4727 | CtWRKY16 | Chr03 | 214 | 645 | 24606.57 | 8.35 | -1.083 | Nucleus |
| evm.model.Hic_asm_9.5063 | CtWRKY17 | Chr03 | 350 | 1053 | 38865.78 | 5.58 | -0.781 | Nucleus |
| evm.model.Hic_asm_7.41 | CtWRKY18 | Chr04 | 205 | 618 | 23114.57 | 8.31 | -0.829 | Nucleus |
| evm.model.Hic_asm_7.225 | CtWRKY19 | Chr04 | 387 | 1164 | 42720.58 | 9.00 | -0.903 | Nucleus |
| evm.model.Hic_asm_7.974 | CtWRKY20 | Chr04 | 327 | 984 | 36107.79 | 6.55 | -0.824 | Nucleus |
| evm.model.Hic_asm_7.1458 | CtWRKY21 | Chr04 | 296 | 891 | 33238.73 | 5.05 | -0.909 | Nucleus |
| evm.model.Hic_asm_7.2452 | CtWRKY22 | Chr04 | 502 | 1509 | 54448.47 | 8.75 | -0.674 | Nucleus |
| evm.model.Hic_asm_11.488 | CtWRKY23 | Chr05 | 477 | 1434 | 51816.29 | 6.97 | -0.858 | Nucleus |
| evm.model.Hic_asm_11.923 | CtWRKY24 | Chr05 | 288 | 867 | 30849.49 | 10.15 | -0.673 | Nucleus |
| evm.model.Hic_asm_11.1186 | CtWRKY25 | Chr05 | 180 | 543 | 20538.54 | 5.96 | -1.005 | Nucleus |
| evm.model.Hic_asm_11.2929 | CtWRKY26 | Chr05 | 216 | 651 | 23899.45 | 9.89 | -0.661 | Nucleus |
| evm.model.Hic_asm_11.3421 | CtWRKY27 | Chr05 | 485 | 1458 | 52703.50 | 6.25 | -0.568 | Nucleus |
| evm.model.Hic_asm_11.3479 | CtWRKY28 | Chr05 | 443 | 1332 | 49148.97 | 6.61 | -1.079 | Nucleus |
| evm.model.Hic_asm_11.3941 | CtWRKY29 | Chr05 | 524 | 1575 | 58091.98 | 5.86 | -0.91 | Nucleus |
| evm.model.Hic_asm_11.3988 | CtWRKY30 | Chr05 | 1336 | 4011 | 150954.83 | 7.18 | -0.474 | Nucleus |
| evm.model.Hic_asm_8.1203 | CtWRKY31 | Chr06 | 224 | 675 | 26239.01 | 8.19 | -1.155 | Nucleus |
| evm.model.Hic_asm_8.5158 | CtWRKY32 | Chr06 | 328 | 987 | 35662.40 | 9.69 | -0.551 | Nucleus |
| evm.model.Hic_asm_8.5326 | CtWRKY33 | Chr06 | 399 | 1200 | 43371.53 | 8.61 | -0.555 | Chloroplast |
| evm.model.Hic_asm_2.97 | CtWRKY34 | Chr07 | 563 | 1692 | 61450.58 | 7.66 | -0.872 | Nucleus |
| evm.model.Hic_asm_2.460 | CtWRKY35 | Chr07 | 430 | 1293 | 47556.29 | 6.44 | -0.99 | Nucleus |
| evm.model.Hic_asm_2.513_evm.model.Hic_asm_2.514 | CtWRKY36 | Chr07 | 522 | 1569 | 57002.67 | 5.48 | -0.778 | Nucleus |
| evm.model.Hic_asm_2.2012 | CtWRKY37 | Chr07 | 344 | 1035 | 38559.67 | 5.58 | -0.679 | Nucleus |
| evm.model.Hic_asm_2.2074 | CtWRKY38 | Chr07 | 244 | 735 | 27017.61 | 4.92 | -0.762 | Nucleus |
| evm.model.Hic_asm_2.2621 | CtWRKY39 | Chr07 | 206 | 621 | 23598.62 | 6.59 | -0.708 | Nucleus |
| evm.model.Hic_asm_2.3904 | CtWRKY40 | Chr07 | 517 | 1554 | 56413.83 | 7.02 | -0.921 | Nucleus |
| evm.model.Hic_asm_2.4209 | CtWRKY41 | Chr07 | 189 | 570 | 21150.49 | 7.64 | -0.858 | Nucleus |
| evm.model.Hic_asm_3.109 | CtWRKY42 | Chr08 | 301 | 906 | 33095.40 | 5.60 | -0.742 | Nucleus |
| evm.model.Hic_asm_3.313 | CtWRKY43 | Chr08 | 183 | 552 | 21067.30 | 5.77 | -0.776 | Nucleus |
| evm.model.Hic_asm_3.316 | CtWRKY44 | Chr08 | 308 | 927 | 34242.72 | 6.31 | -0.765 | Nucleus |
| evm.model.Hic_asm_3.326 | CtWRKY45 | Chr08 | 263 | 792 | 30062.02 | 8.94 | -0.65 | Nucleus |
| evm.model.Hic_asm_3.327 | CtWRKY46 | Chr08 | 325 | 978 | 36213.46 | 6.25 | -0.674 | Nucleus |
| evm.model.Hic_asm_3.1017 | CtWRKY47 | Chr08 | 412 | 1239 | 46207.72 | 9.34 | -0.851 | Nucleus |
| evm.model.Hic_asm_3.5176 | CtWRKY48 | Chr08 | 1196 | 3591 | 135589.79 | 5.97 | -0.397 | Peroxisome |
| evm.model.Hic_asm_4.461 | CtWRKY49 | Chr09 | 554 | 1665 | 61604.53 | 6.66 | -0.958 | Nucleus |
| evm.model.Hic_asm_4.1108 | CtWRKY50 | Chr09 | 304 | 915 | 33808.20 | 9.83 | -0.767 | Nucleus |
| evm.model.Hic_asm_4.2954 | CtWRKY51 | Chr09 | 559 | 1680 | 61330.86 | 7.56 | -0.867 | Nucleus |
| evm.model.Hic_asm_4.3177 | CtWRKY52 | Chr09 | 208 | 627 | 23013.62 | 8.86 | -0.624 | Cytoplasm |
| evm.model.Hic_asm_4.3178 | CtWRKY53 | Chr09 | 344 | 1035 | 37088.76 | 6.85 | -0.414 | Nucleus |
| evm.model.Hic_asm_4.3902 | CtWRKY54 | Chr09 | 363 | 1092 | 39207.99 | 9.65 | -0.57 | Nucleus |
| evm.model.Hic_asm_4.4506 | CtWRKY55 | Chr09 | 339 | 1020 | 37349.15 | 8.91 | -1.039 | Nucleus |
| evm.model.Hic_asm_5.133 | CtWRKY56 | Chr10 | 215 | 648 | 24816.47 | 4.94 | -0.972 | Nucleus |
| evm.model.Hic_asm_5.688 | CtWRKY57 | Chr10 | 514 | 1545 | 57494.50 | 7.27 | -0.872 | Nucleus |
| evm.model.Hic_asm_5.1608 | CtWRKY58 | Chr10 | 702 | 2109 | 78016.33 | 5.42 | -0.801 | Nucleus |
| evm.model.Hic_asm_5.1974 | CtWRKY59 | Chr10 | 189 | 570 | 21736.20 | 7.66 | -0.828 | Nucleus |
| evm.model.Hic_asm_5.2328 | CtWRKY60 | Chr10 | 375 | 1128 | 42241.85 | 6.53 | -0.771 | Nucleus |
| evm.model.Hic_asm_5.2329 | CtWRKY61 | Chr10 | 270 | 813 | 30566.19 | 8.58 | -0.817 | Nucleus |
| evm.model.Hic_asm_5.2334 | CtWRKY62 | Chr10 | 257 | 774 | 29057.50 | 6.71 | -0.786 | Nucleus |
| evm.model.Hic_asm_5.3186 | CtWRKY63 | Chr10 | 274 | 825 | 30690.30 | 5.11 | -0.63 | Nucleus |
| evm.model.Hic_asm_5.3681 | CtWRKY64 | Chr10 | 451 | 1356 | 51284.10 | 5.78 | -1.068 | Nucleus |
| evm.model.Hic_asm_5.3957 | CtWRKY65 | Chr10 | 378 | 1137 | 43441.65 | 6.32 | -0.779 | Nucleus |
| evm.model.Hic_asm_5.3962 | CtWRKY66 | Chr10 | 296 | 891 | 33904.56 | 9.54 | -0.789 | Nucleus |
| evm.model.Hic_asm_5.3980 | CtWRKY67 | Chr10 | 350 | 1053 | 39964.65 | 6.17 | -0.805 | Nucleus |
| evm.model.Hic_asm_5.3986 | CtWRKY68 | Chr10 | 395 | 1188 | 44932.91 | 5.25 | -0.779 | Nucleus |
| evm.model.Hic_asm_5.3998 | CtWRKY69 | Chr10 | 374 | 1125 | 42471.30 | 5.49 | -0.756 | Nucleus |
| evm.model.Hic_asm_5.4012 | CtWRKY70 | Chr10 | 323 | 972 | 36507.73 | 6.91 | -0.391 | Nucleus |
| evm.model.Hic_asm_5.4019 | CtWRKY71 | Chr10 | 313 | 942 | 35177.29 | 6.10 | -0.735 | Nucleus |
| evm.model.Hic_asm_6.354 | CtWRKY72 | Chr11 | 329 | 990 | 35758.52 | 9.59 | -0.584 | Nucleus |
| evm.model.Hic_asm_6.664 | CtWRKY73 | Chr11 | 493 | 1482 | 53429.49 | 8.35 | -0.743 | Nucleus |
| evm.model.Hic_asm_6.2962 | CtWRKY74 | Chr11 | 317 | 954 | 35550.47 | 5.66 | -0.703 | Nucleus |
| evm.model.Hic_asm_6.2963 | CtWRKY75 | Chr11 | 266 | 801 | 30357.80 | 5.55 | -0.744 | Mitochondrion |
| evm.model.Hic_asm_6.3743 | CtWRKY76 | Chr11 | 252 | 759 | 28270.30 | 5.56 | -0.975 | Nucleus |
| evm.model.Hic_asm_1.629 | CtWRKY77 | Chr12 | 237 | 714 | 26681.39 | 8.18 | -1.054 | Nucleus |
| evm.model.Hic_asm_1.1723 | CtWRKY78 | Chr12 | 167 | 504 | 19130.99 | 5.24 | -1.069 | Nucleus |
| evm.model.Hic_asm_1.2116 | CtWRKY79 | Chr12 | 483 | 1452 | 53952.57 | 6.58 | -1.092 | Nucleus |
| evm.model.Hic_asm_1.3085 | CtWRKY80 | Chr12 | 431 | 1296 | 47465.12 | 6.49 | -0.756 | Nucleus |
| evm.model.Hic_asm_1.3280 | CtWRKY81 | Chr12 | 282 | 849 | 30446.32 | 9.98 | -0.579 | Nucleus |
| evm.model.Hic_asm_1.3988 | CtWRKY82 | Chr12 | 354 | 1065 | 39531.83 | 9.70 | -0.773 | Nucleus |

Table S2. Regular expressions of conserved motifs from CtWRKY proteins

| **Motif** | **E-value** | **Sites** | **Width** | **Multilevel consensus sequence** |
| --- | --- | --- | --- | --- |
| **1** | 2.7e-1084 | 80 | 19 | YRWRKYGQKVIKGSPHPRS |
| **2** | 1.6e-1226 | 77 | 29 | AGCPVRKQVZRASDDPSMVITTYEGKHNH |
| **3** | 3.6e-344 | 17 | 29 | GYNWRKYGQKQVKGSEYPRSYYKCTHPNC |
| **4** | 4.6e-308 | 37 | 21 | TIREPRVVVQTRSEVDILDDG |
| **5** | 9.3e-287 | 81 | 6 | YYRCTS |
| **6** | 1.5e-221 | 18 | 29 | PVKKKVERSLDGHITEIVYKGQHBHPKPQ |
| **7** | 1.5e-102 | 17 | 29 | RKKAIZELTQGQELTKKLRELLRRSNNSD |
| **8** | 8.1e-077 | 14 | 22 | SVDGLVGQILGMFDBTLSILTS |
| **9** | 5.9e-089 | 19 | 21 | SKGRRGCYKRRKTRDKRTKIV |
| **10** | 9.0e-080 | 11 | 29 | LEKMNEENQRLREMLTQVMEBYNTLQMHL |

Table S3. Ka/Ks analysis of CtWRKY genes of safflower

| **Seq 1** | **Seq 2** | **Ka** | **Ks** | **Ka/Ks** | **Duplication type** | **Purify selection** |
| --- | --- | --- | --- | --- | --- | --- |
| CtWRKY43 | CtWRKY44 | 0.354081228 | 1.167049879 | 0.303398539 | Tandem | Yes |
| CtWRKY45 | CtWRKY46 | 0.708169327 | NaN | NaN | Tandem | / |
| CtWRKY52 | CtWRKY53 | 0.810640754 | 2.968367723 | 0.273093104 | Tandem | Yes |
| CtWRKY67 | CtWRKY68 | 0.125999147 | 0.20235456 | 0.622665221 | Tandem | Yes |
| CtWRKY74 | CtWRKY75 | 0.065867184 | 0.233734765 | 0.281803111 | Tandem | Yes |
| CtWRKY1 | CtWRKY19 | 0.428864582 | 1.996661749 | 0.214790804 | Segmental | Yes |
| CtWRKY1 | CtWRKY73 | 0.271818256 | 1.69434835 | 0.16042643 | Segmental | Yes |
| CtWRKY4 | CtWRKY21 | 0.162180683 | 1.266860364 | 0.128017805 | Segmental | Yes |
| CtWRKY5 | CtWRKY13 | 0.434734483 | NaN | NaN | Segmental | / |
| CtWRKY5 | CtWRKY22 | 0.229989726 | 1.278561069 | 0.17988169 | Segmental | Yes |
| CtWRKY7 | CtWRKY18 | 0.244725004 | 1.579780818 | 0.154910732 | Segmental | Yes |
| CtWRKY7 | CtWRKY41 | 0.231596646 | 2.144652629 | 0.107987952 | Segmental | Yes |
| CtWRKY9 | CtWRKY76 | 0.195105555 | 0.924170384 | 0.211114269 | Segmental | Yes |
| CtWRKY12 | CtWRKY28 | 0.584116728 | NaN | NaN | Segmental | / |
| CtWRKY12 | CtWRKY34 | 0.1788227 | 0.696210449 | 0.256851503 | Segmental | Yes |
| CtWRKY12 | CtWRKY52 | 0.174108257 | 0.927473416 | 0.187723178 | Segmental | Yes |
| CtWRKY13 | CtWRKY27 | 0.237864673 | 0.965402594 | 0.246389096 | Segmental | Yes |
| CtWRKY13 | CtWRKY36 | 0.231494948 | 0.861842269 | 0.268604774 | Segmental | Yes |
| CtWRKY17 | CtWRKY74 | 0.363882387 | 1.104128543 | 0.329565238 | Segmental | Yes |
| CtWRKY18 | CtWRKY41 | 0.228990747 | 1.225708482 | 0.186823172 | Segmental | Yes |
| CtWRKY19 | CtWRKY40 | 0.221928191 | 1.148675069 | 0.193203628 | Segmental | Yes |
| CtWRKY19 | CtWRKY73 | 0.433285472 | 2.641057914 | 0.164057543 | Segmental | Yes |
| CtWRKY20 | CtWRKY8 | 0.796549567 | 2.268513072 | 0.351132897 | Segmental | Yes |
| CtWRKY22 | CtWRKY30 | 0.904300253 | 2.151928084 | 0.420227915 | Segmental | Yes |
| CtWRKY23 | CtWRKY80 | 0.238103492 | 0.716459135 | 0.332333668 | Segmental | Yes |
| CtWRKY24 | CtWRKY81 | 0.202396069 | 0.656784577 | 0.308162031 | Segmental | Yes |
| CtWRKY27 | CtWRKY36 | 0.324851143 | 1.100277094 | 0.295244848 | Segmental | Yes |
| CtWRKY28 | CtWRKY35 | 0.227402297 | 0.769599537 | 0.295481332 | Segmental | Yes |
| CtWRKY32 | CtWRKY54 | 0.16474367 | 1.128058048 | 0.146041837 | Segmental | Yes |
| CtWRKY32 | CtWRKY72 | 0.304567376 | 1.935277398 | 0.1573766 | Segmental | Yes |
| CtWRKY34 | CtWRKY52 | 0.21603846 | 0.988034599 | 0.218654752 | Segmental | Yes |
| CtWRKY38 | CtWRKY42 | 0.245373445 | 1.007645021 | 0.243511792 | Segmental | Yes |
| CtWRKY39 | CtWRKY46 | 0.346450848 | 2.529901331 | 0.136942435 | Segmental | Yes |
| CtWRKY40 | CtWRKY73 | 0.365900174 | NaN | NaN | Segmental | / |
| CtWRKY45 | CtWRKY60 | 0.355034591 | 1.161799338 | 0.305590285 | Segmental | Yes |
| CtWRKY49 | CtWRKY57 | 0.251648153 | 1.438526153 | 0.174934708 | Segmental | Yes |
| CtWRKY50 | CtWRKY82 | 0.155536598 | 0.884946864 | 0.17575812 | Segmental | Yes |
| CtWRKY54 | CtWRKY72 | 0.342647081 | 2.780797877 | 0.123218981 | Segmental | Yes |
| CtWRKY64 | CtWRKY79 | 0.308396655 | 0.998323349 | 0.308914597 | Segmental | Yes |

Table S4. The original data of expression profiles of *CtWRKY1-82* genes in 10 tissues

| **Gnen name** | **Seed** | **Leaf** | **Flower** | | **Root**  **(high light)** | **Stem**  **(high light)** | **Leaf**  **(high light)** | **Flower**  **(medium light)** | **Leaf (EBR)** |
| --- | --- | --- | --- | --- | --- | --- | --- | --- | --- |
| CtWRKY1 | 0.00 | 1.77 | 0.07 | 0.00 | | 0.58 | 0.51 | 0.08 | 5.37 |
| CtWRKY10 | 0.00 | 11.17 | 0.34 | 3.33 | | 0.48 | 16.85 | 0.00 | 1.85 |
| CtWRKY11 | 6.00 | 8.77 | 8.56 | 32.48 | | 28.06 | 18.34 | 16.21 | 21.39 |
| CtWRKY12 | 6.59 | 3.95 | 2.08 | 4.64 | | 5.80 | 3.09 | 2.04 | 6.03 |
| CtWRKY13 | 3.39 | 16.75 | 0.82 | 19.48 | | 3.39 | 17.91 | 0.67 | 10.34 |
| CtWRKY14 | 0.11 | 0.21 | 0.16 | 12.37 | | 9.37 | 0.52 | 0.46 | 0.71 |
| CtWRKY15 | 0.10 | 161.78 | 8.61 | 16.34 | | 94.61 | 34.26 | 7.29 | 21.12 |
| CtWRKY16 | 0.00 | 2.08 | 10.29 | 9.39 | | 8.52 | 17.96 | 5.15 | 4.59 |
| CtWRKY17 | 0.61 | 53.25 | 2.72 | 28.81 | | 71.87 | 4.93 | 0.88 | 2.88 |
| CtWRKY18 | 0.00 | 66.91 | 25.25 | 19.19 | | 4.01 | 17.11 | 1.72 | 2.50 |
| CtWRKY19 | 0.05 | 0.32 | 0.63 | 2.10 | | 0.32 | 0.94 | 0.20 | 0.29 |
| CtWRKY2 | 0.05 | 8.07 | 1.93 | 12.61 | | 3.42 | 11.54 | 0.23 | 0.46 |
| CtWRKY20 | 1.07 | 0.20 | 0.15 | 6.16 | | 8.11 | 0.66 | 0.74 | 0.38 |
| CtWRKY21 | 0.12 | 7.30 | 4.18 | 31.23 | | 2.64 | 6.03 | 0.40 | 23.66 |
| CtWRKY22 | 0.32 | 22.94 | 97.91 | 6.56 | | 17.21 | 7.52 | 14.65 | 10.81 |
| CtWRKY23 | 10.38 | 6.02 | 10.47 | 3.18 | | 7.76 | 6.16 | 7.41 | 12.44 |
| CtWRKY24 | 14.40 | 32.00 | 29.77 | 103.06 | | 74.31 | 21.76 | 16.34 | 51.67 |
| CtWRKY25 | 0.46 | 36.06 | 1.84 | 9.91 | | 21.63 | 28.20 | 0.35 | 12.99 |
| CtWRKY26 | 0.56 | 2.05 | 3.20 | 8.34 | | 22.79 | 6.85 | 5.88 | 7.95 |
| CtWRKY27 | 0.00 | 3.76 | 0.11 | 5.86 | | 0.23 | 5.60 | 0.00 | 0.85 |
| CtWRKY28 | 12.34 | 12.77 | 14.02 | 7.59 | | 21.74 | 12.30 | 12.94 | 18.08 |
| CtWRKY29 | 0.00 | 0.81 | 0.27 | 0.10 | | 0.18 | 1.08 | 0.00 | 0.32 |
| CtWRKY3 | 0.09 | 0.04 | 0.13 | 0.11 | | 2.38 | 0.06 | 0.24 | 0.20 |
| CtWRKY30 | 1.88 | 18.83 | 33.75 | 1.83 | | 16.61 | 20.49 | 5.77 | 48.95 |
| CtWRKY31 | 0.35 | 0.24 | 0.12 | 14.11 | | 27.19 | 1.03 | 0.00 | 0.89 |
| CtWRKY32 | 0.82 | 7.27 | 5.17 | 13.96 | | 13.68 | 5.76 | 1.65 | 2.13 |
| CtWRKY33 | 2.61 | 3.42 | 5.50 | 6.99 | | 9.65 | 2.37 | 1.19 | 2.20 |
| CtWRKY34 | 4.13 | 11.30 | 11.25 | 19.00 | | 22.80 | 13.37 | 9.67 | 27.26 |
| CtWRKY35 | 22.13 | 19.55 | 18.18 | 11.67 | | 25.40 | 19.08 | 14.24 | 28.25 |
| CtWRKY36 | 0.13 | 4.82 | 0.04 | 4.09 | | 0.82 | 2.49 | 1.01 | 0.04 |
| CtWRKY37 | 0.00 | 7.40 | 0.11 | 0.08 | | 2.18 | 0.12 | 0.00 | 0.38 |
| CtWRKY38 | 0.10 | 0.53 | 0.26 | 1.29 | | 14.41 | 0.91 | 0.09 | 0.15 |
| CtWRKY39 | 0.00 | 0.00 | 0.00 | 0.00 | | 0.00 | 0.00 | 0.00 | 0.00 |
| CtWRKY4 | 0.00 | 0.19 | 0.43 | 10.62 | | 0.96 | 0.60 | 2.40 | 1.40 |
| CtWRKY40 | 6.37 | 11.08 | 21.26 | 5.51 | | 4.06 | 7.10 | 2.54 | 9.61 |
| CtWRKY41 | 0.16 | 25.54 | 17.80 | 4.69 | | 6.94 | 14.78 | 5.01 | 44.59 |
| CtWRKY42 | 0.14 | 0.12 | 0.00 | 3.35 | | 27.01 | 0.26 | 0.00 | 0.10 |
| CtWRKY43 | 0.00 | 0.00 | 0.00 | 0.36 | | 0.00 | 0.00 | 0.00 | 0.00 |
| CtWRKY44 | 0.05 | 79.47 | 79.36 | 62.11 | | 117.49 | 46.96 | 8.70 | 16.66 |
| CtWRKY45 | 0.00 | 26.16 | 0.13 | 2.28 | | 1.40 | 31.61 | 0.08 | 8.16 |
| CtWRKY46 | 0.00 | 2.55 | 0.28 | 0.89 | | 0.26 | 0.98 | 0.00 | 0.22 |
| CtWRKY47 | 6.30 | 2.92 | 4.77 | 2.04 | | 3.72 | 5.73 | 4.79 | 10.23 |
| CtWRKY48 | 0.04 | 0.45 | 0.08 | 0.13 | | 1.03 | 0.56 | 0.51 | 0.57 |
| CtWRKY49 | 4.86 | 40.08 | 100.13 | 17.47 | | 15.63 | 14.46 | 9.24 | 13.12 |
| CtWRKY5 | 0.48 | 44.35 | 41.24 | 24.53 | | 13.81 | 9.38 | 0.86 | 1.48 |
| CtWRKY50 | 11.83 | 9.89 | 4.79 | 24.70 | | 20.70 | 22.53 | 6.58 | 10.16 |
| CtWRKY51 | 0.10 | 0.25 | 1.25 | 1.57 | | 0.03 | 0.07 | 0.00 | 0.02 |
| CtWRKY52 | 6.29 | 2.32 | 8.48 | 0.52 | | 4.63 | 1.64 | 5.69 | 3.73 |
| CtWRKY53 | 0.98 | 0.68 | 3.60 | 0.61 | | 3.04 | 1.10 | 2.72 | 4.95 |
| CtWRKY54 | 2.01 | 64.96 | 39.38 | 105.29 | | 66.23 | 12.17 | 31.92 | 24.07 |
| CtWRKY55 | 5.01 | 373.85 | 223.33 | 31.50 | | 83.78 | 27.49 | 32.42 | 35.92 |
| CtWRKY56 | 0.66 | 7.47 | 1.62 | 1.99 | | 6.75 | 18.24 | 1.82 | 56.48 |
| CtWRKY57 | 0.28 | 63.47 | 46.57 | 16.94 | | 23.50 | 15.92 | 1.83 | 12.33 |
| CtWRKY58 | 1.02 | 4.23 | 1.39 | 5.74 | | 13.41 | 5.50 | 4.99 | 2.30 |
| CtWRKY59 | 0.14 | 0.00 | 0.00 | 1.05 | | 0.00 | 0.00 | 0.00 | 0.00 |
| CtWRKY6 | 0.00 | 0.00 | 0.14 | 0.00 | | 0.29 | 0.00 | 0.00 | 0.00 |
| CtWRKY60 | 1.73 | 4.54 | 1.74 | 2.72 | | 8.89 | 4.15 | 2.98 | 3.27 |
| CtWRKY61 | 9.23 | 63.32 | 18.76 | 17.20 | | 111.71 | 21.40 | 1.45 | 27.31 |
| CtWRKY62 | 1.57 | 3.63 | 0.48 | 1.12 | | 15.30 | 1.80 | 0.74 | 1.52 |
| CtWRKY63 | 0.00 | 0.00 | 0.00 | 0.89 | | 0.16 | 0.00 | 0.00 | 0.28 |
| CtWRKY64 | 0.00 | 0.10 | 0.00 | 0.00 | | 0.11 | 0.04 | 0.00 | 0.00 |
| CtWRKY65 | 0.00 | 0.45 | 0.10 | 8.07 | | 1.16 | 2.21 | 0.00 | 0.05 |
| CtWRKY66 | 0.00 | 0.00 | 0.00 | 0.19 | | 0.40 | 0.00 | 0.00 | 0.00 |
| CtWRKY67 | 0.00 | 25.37 | 0.66 | 7.77 | | 15.37 | 6.17 | 0.00 | 0.98 |
| CtWRKY68 | 0.00 | 0.11 | 0.08 | 0.11 | | 0.00 | 0.00 | 0.00 | 0.12 |
| CtWRKY69 | 0.00 | 0.11 | 0.00 | 0.00 | | 0.12 | 0.00 | 0.00 | 0.00 |
| CtWRKY7 | 1.53 | 30.39 | 22.47 | 34.92 | | 9.61 | 13.34 | 18.39 | 31.24 |
| CtWRKY70 | 0.00 | 0.00 | 0.00 | 0.00 | | 0.00 | 0.00 | 0.00 | 0.00 |
| CtWRKY71 | 0.00 | 15.57 | 2.09 | 11.95 | | 11.13 | 48.22 | 1.76 | 2.12 |
| CtWRKY72 | 22.68 | 70.62 | 42.88 | 143.41 | | 88.79 | 43.19 | 25.99 | 42.16 |
| CtWRKY73 | 22.48 | 56.53 | 40.54 | 11.37 | | 28.05 | 49.84 | 22.04 | 74.64 |
| CtWRKY74 | 37.45 | 212.86 | 10.46 | 206.77 | | 185.88 | 2.47 | 12.13 | 52.93 |
| CtWRKY75 | 14.72 | 163.65 | 15.89 | 161.12 | | 143.54 | 2.18 | 8.24 | 76.20 |
| CtWRKY76 | 0.07 | 3.28 | 5.41 | 10.87 | | 7.81 | 1.80 | 4.02 | 3.49 |
| CtWRKY77 | 9.45 | 8.21 | 14.80 | 3.56 | | 11.83 | 7.17 | 17.44 | 15.73 |
| CtWRKY78 | 0.00 | 22.56 | 0.00 | 2.09 | | 0.00 | 9.25 | 0.00 | 1.46 |
| CtWRKY79 | 23.06 | 137.15 | 46.51 | 37.71 | | 134.02 | 12.08 | 8.88 | 82.00 |
| CtWRKY8 | 0.00 | 0.02 | 0.18 | 1.02 | | 0.06 | 0.00 | 0.00 | 0.00 |
| CtWRKY80 | 3.89 | 5.91 | 2.54 | 4.82 | | 9.69 | 8.88 | 7.11 | 6.06 |
| CtWRKY81 | 23.06 | 69.20 | 12.99 | 225.44 | | 85.05 | 16.41 | 9.73 | 57.28 |
| CtWRKY82 | 8.54 | 10.64 | 7.49 | 33.41 | | 25.14 | 8.77 | 10.64 | 27.97 |
| CtWRKY9 | 10.71 | 6.13 | 8.48 | 6.96 | | 3.12 | 6.03 | 3.67 | 3.77 |

Table S5. Primers used in the paper

| **Constructions** | **Primer sequences** |
| --- | --- |
| **Primers for qRT-PCR analysis** |  |
| EF-1a-F | TCAGCATTGTCGTCATCGGA |
| EF-1a-R | ACGTTCGATCACACGCTTGTC |
| Actin-F | TACCGGAATGGTTAAGGCTGG |
| Actin-R | CATCCTTTTGGCCCATTCCT |
| CtWRKY9-F | GTACGGCCAAAAAGCTGTCA |
| CtWRKY9-R | TGGGGCATGGGTGTTTATGG |
| CtWRKY15-F | TGCTAATCATCCAAGGGCGT |
| CtWRKY15-R | GCTTTTGATAGCTGAGCGGC |
| CtWRKY17-F | AAAACAAGTGCAGCGAACCG |
| CtWRKY17-R | CGAGAGCATGTCGACGGATT |
| CtWRKY37-F | TCAGAAGGTTGCCTTGCGAC |
| CtWRKY37-R | TGGCGGTGATGACATTGGTA |
| CtWRKY54-F | ACCAGAACCAGAACCATCCG |
| CtWRKY54-R | AGCAGCGAAATTGATGGTGG |
| CtWRKY55-F | GGCCTCTTGGTGAAGGATGG |
| CtWRKY55-R | TTGTGCTCGCCTTCGTAAGT |
| CtWRKY72-F | AGTAATCAAGCGGCGGTGTT |
| CtWRKY72-R | AACAACGGAGGCTTTCCA |
